# Synthesis of New Hydrated Geranylphenols and *in Vitro* Antifungal Activity against *Botrytis cinerea*

**DOI:** 10.3390/ijms17060840

**Published:** 2016-06-03

**Authors:** Mauricio Soto, Luis Espinoza, María I. Chávez, Katy Díaz, Andrés F. Olea, Lautaro Taborga

**Affiliations:** 1 Departamento de Química, Universidad Técnica Federico Santa María, Valparaíso 2340000, Chile; mauricio.sotoc.13@sansano.usm.cl (M.S.); luis.espinozac@usm.cl (L.E.); maria.chavez@usm.cl (M.I.C.); katy.diaz@usm.cl (K.D.); 2 Instituto de Ciencias Químicas Aplicadas, Facultad de Ingeniería, Universidad Autónoma de Chile, Santiago 8910339, Chile; andres.olea@uautonoma.cl

**Keywords:** geranylated phenol derivatives, hydrated geranyl, synthesis, structural elucidation, growth inhibition effect, *Botrytis cinerea*, fungicide

## Abstract

Geranylated hydroquinones and other geranylated compounds isolated from *Aplydium* species have shown interesting biological activities. This fact has prompted a number of studies where geranylated phenol derivatives have been synthesized in order to assay their bioactivities. In this work, we report the synthesis of a series of new hydrated geranylphenols using two different synthetic approaches and their inhibitory effects on the mycelial growth of *Botrytis cinerea*. Five new hydrated geranylphenols were obtained by direct coupling reaction between geraniol and phenol in dioxane/water and using BF_3_·Et_2_O as the catalyst or by the reaction of a geranylated phenol with BF_3_·Et_2_O. Two new geranylated quinones were also obtained. The synthesis and structural elucidation of all new compounds is presented. All hydrated geranylphenols efficiently inhibit the mycelial growth of *B. cinerea*. Their activity is higher than that observed for non-hydrated compounds. These results indicate that structural modification on the geranyl chain brings about an enhancement of the inhibition effect of geranylated phenol derivatives.

## 1. Introduction

**Figure 1:**
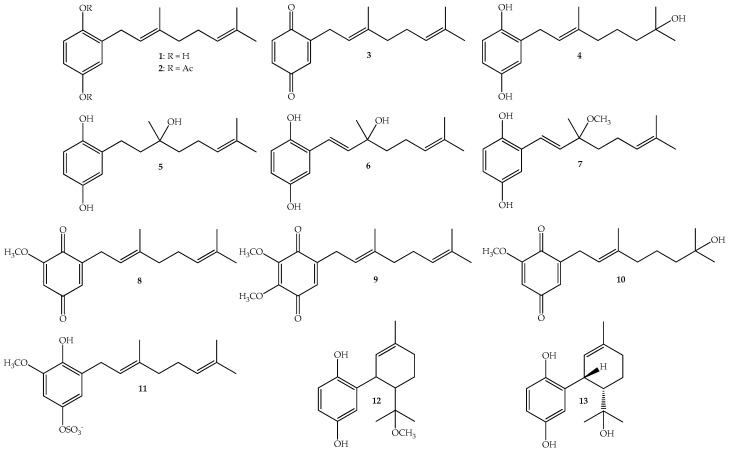
Structure of some naturally-occurring linear geranylated hydroquinones/quinones **1**–**11,** and cyclodiprenyl meroterpenes **12**–**13**.

The subgroups of linear geranylated quinones, geranylated hydroquinones and meroterpenes are represented by an important number of metabolites isolated from ascidians belonging to the genus *Aplydium* [1,2]. The first biologically active tunicate metabolites were 2-geranylhydroquinone (**1**) and 2-geranyl hydroquinone diacetate (**2**) (Figure 1), isolated from *Aplydium* sp. and *Phacelia crenulata* [3,4] and *Pyrola japonica* [5], respectively, and later found in many others *Aplydium* species. It has been shown that these compounds exhibit antitumoral activity [6]. Additionally, several linear quinones/hydroquinones carrying a geranyl type side chain (Compounds **1**, **3**–**11**; Figure 1) have been obtained from diverse *Aplydium* species [7,8,9,10,11,12]. Compound **1** and 2-geranylbenzoquinone (**3**) exhibit interesting and various biological activities [3,8,13,14,15,16,17], whereas Compounds **5** and **6** show antioxidant activities [8], and Compound **4** shows cytotoxicity activity against P-388 mouse lymphoma suspension culture [18]. Additionally, linear geranylmethoxyphenol/acetates derivatives isolated from *Phacelia ixodes* [19] are cytotoxic, allergenic and insecticidal.

On the other hand, the anticancer properties, both *in vitro* and *in vivo*, of a group of prenylated quinones, *i.e.*, 3-demethylubiquinone Q2 (**9**) and its synthetic analogs, were studied as a function of their molecular structure [20,21]. The results indicate that **9** and its derivatives are able to inhibit the growth of human cancer cell lines and of the solid Ehrlich carcinoma in mice by inducing apoptosis in cancer cells. The anticancer activity of this structural-related family of compounds depends on the length of the polyprenyl chain and on the position of the methoxyl groups in the quinone part of the molecule [20]. The most effective compounds were those having a side chain of the geranyl type (two isoprene units) and one methoxyl group at the para-position to the geranyl chain.

Cyclodiprenyl hydroquinones, such as methoxyconidiol (**12**) and conitriol (**13**), have been isolated from *Aplidium aff. densum* [22] and *Aplidium conicum* [9], respectively. Methoxyconidiol and its methoxy derivative were synthesized, and their biological activities on human cancer cell lines and sea urchin embryos were assessed [23]. A detailed description of the isolation and biological activities of these and other structures of natural prenylquinones, hydroquinones and meroterpenes can be found in [24,25].

Thus, the interesting biological activity shown by **1** and other prenyl derivatives [17] (see Figure 1) has prompted us to undertake the synthesis of a significant number of linear geranylated phenols, including Compounds **1**–**3** and some geranylated methoxyphenyl/acetate analogs [26,27,28,29,30,31,32], in order to evaluate the *in vitro* cytotoxic activity on some cancer lines and the inhibitory effects on the mycelial growth of plant pathogen *Botrytis cinerea* [29,30,31]. The latter is a facultative phytopathogenic fungus that attacks the flowers, fruits, leaves and stems of more than 200 plant species [33]. In Chile, there is a high incidence of this fungus, and its control by commercial fungicides (dicarboximides and benzimidazoles) is becoming more ineffective due to the appearance of highly resistant strains [34,35]. Thus, an increasing number of metabolites isolated from plants, hemisynthetic and synthetic products have been studied as an alternative to chemical fungicide [29,36,37,38]. Previous work has shown that the anti-fungal activity of geranylated phenols is mainly determined by the presence of the geranyl chain and substitution on the aromatic ring [29,31]. However, there are no data regarding the fungicide activity of compounds carrying a modified geranyl chain, e.g., hydrated geranylphenols.

**Figure 2:**
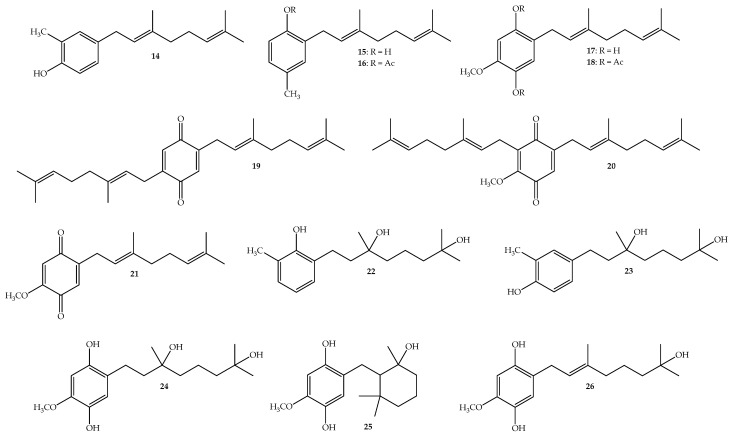
Chemical structures of geranylated phenols (**14**–**18**) geranylated quinones (**19**–**21**) and hydrated geranylphenols derivatives (**22**–**26**) that have been studied in this work.

Therefore, in this research, a study of the inhibitory effects on the mycelial growth of plant pathogen *B. cinerea* of geranylated phenols (Compounds **14**–**18**), geranylated quinones (Compounds **19**–**21**) and hydrated geranylphenols derivatives (Compounds **22**–**26**) (see Figure 2) is reported. The synthesis and structural elucidation of the new compounds (**14**–**18**, **20**, **22**–**26**) is also presented.

## 2. Results and Discussion

### 2.1. Synthesis

Linear geranylated phenols/methoxyphenols have been synthesized by direct coupling of geraniol with the respective phenol or methoxyphenols. This reaction has been studied for many authors, because it is directly related to the synthesis of biologically-active phenolic terpenoids [11,14,20,26,27,28,29,30]. The coupling is commonly carried out in strong mineral acids or aprotic solvents with Lewis acids, e.g., BF_3_·Et_2_O, in dioxane for the synthesis of tocopherols and geranyl and farnesyl analogs of the ubiquinones, *p*-toluenesulfonic acid in CH_2_CI_2_ for the synthesis of cannabigerol and related marihuana constituents [39]. Alternatively, BF_3_·Et_2_O/AgNO_3_ has been used as a catalyst and acetonitrile as a solvent [29,30]. In this work, Compounds **1**, **3**, **14**, **15**, **17** and **19** were synthesized through this reaction, using dioxane as the solvent, BF_3_·Et_2_O as the catalyst and in the presence or absence of a nitrogen atmosphere.

**Scheme 1:**
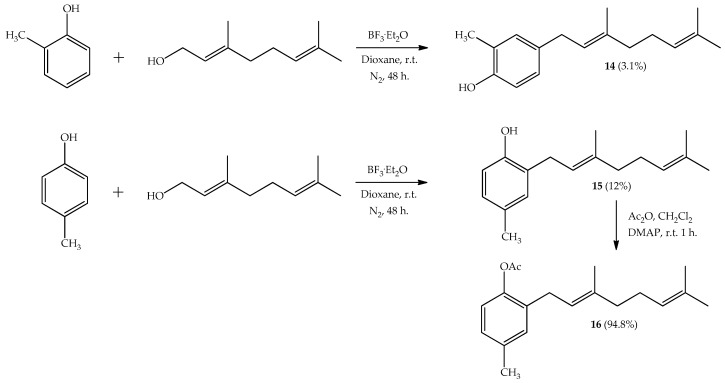
Synthesis of Compounds **14**, **15** and **16**.

Direct coupling between *o*-cresol and *p*-cresol with geraniol under a nitrogen atmosphere leads to Compounds **14** and **15** with 3.1% and 12% yields, respectively (Scheme 1).

**Scheme 2:**
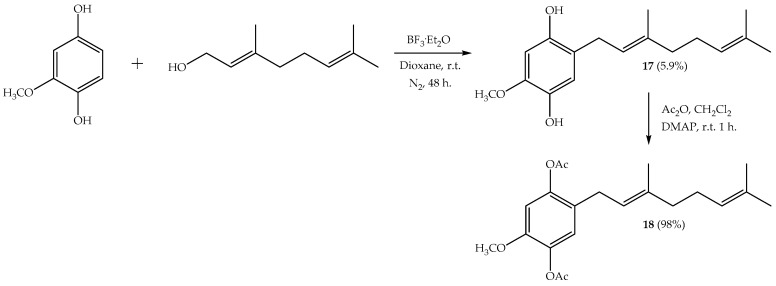
Synthesis of Compounds **17** and **18**.

Following the same synthetic procedure, Compound **17** is obtained as a unique product by coupling between 2-metoxyhydroquinone and geraniol with 5.9% yield (Scheme 2).

Standard acetylation (Ac_2_O/CH_2_Cl_2_/DMAP) of **15** and **17** gives the acetylated derivatives **16** and **18** with 94.8% and 98% yields, respectively.

**Scheme 3:**
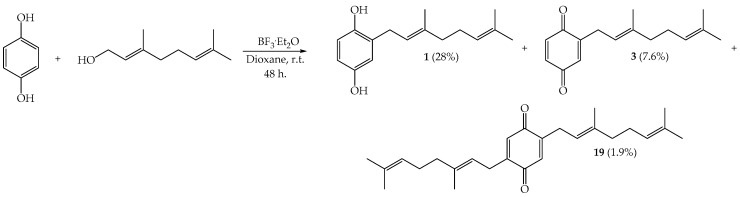
Synthesis of Compounds **1**, **3** and **19**.

**Scheme 4:**
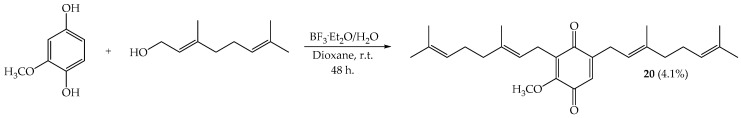
Synthesis of Compound **20**.

**Scheme 5:**
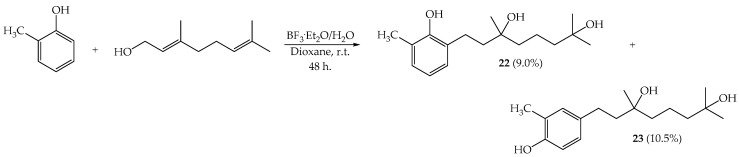
Synthesis of Compounds **22** and **23** by coupling of *o*-cresol and geraniol, in the presence of water and the absence of a nitrogen atmosphere.

In the search for a synthetic pathway to obtain hydrated geranylphenols, we have attempted two different approaches. In the first one, we explore the possibility of obtaining both geranylated quinones and hydrated geranylphenols in a one-pot synthesis. It has been reported that some hydrated geranylorcinols have been obtained as minor products in the coupling reaction of orcinol and geraniol in the presence of 1% aqueous oxalic acid at 80 °C [40]. Therefore, in this approach, the coupling reaction is carried out under air using dioxane as the solvent, BF_3_·Et_2_O as the catalyst and small amounts of added water. Interestingly, the obtained products depend on the chemical nature of the reacting phenol. Thus, coupling between geraniol and 1,4-hydroquinone leads to monogeranylated hydroquinone **1** and quinone **2**, as well as digeranylated quinone **19** (Scheme 3); whereas, coupling between 2-metoxyhydroquinone and geraniol gives the disubstituted quinone **20** as the only product (Scheme 4). Finally, hydrated geranylphenols **22** and **23** were obtained in the coupling reaction between *o*-cresol and geraniol (Scheme 5).

In this reaction, Compounds **1**, **3** and **19** were obtained with 28.0%, 7.6% and 1.9% yields, respectively. When this coupling is carried out in the presence of a nitrogen atmosphere and with no added water, Compound **1** is obtained as the exclusive product [26]. Recently, this reaction has been performed at higher temperatures using aluminum phenoxide as the catalyst, and a completely different pattern of products has been reported. Compound **1** and a mixture of digeranylated quinones (**19** and di-ortho-geranylated quinone) were obtained with 40% and 27% yields, respectively, but Compound **3** was not identified [41].

On the other hand, a methoxy substitution in the hydroquinone induces a complete change in the product distribution, *i.e.*, Compound **20** is obtained with 4.1% yield.

It is worth mentioning that geranylated quinones (**3**, **19**, **20**) are formed only by coupling geraniol with 1,4-dihydroxybenzene systems. Probably, the oxidation to 1,4-quinone is enhanced by the redox properties of hydroquinone compounds.

Finally, in the coupling of geraniol with *o*-cresol, hydrated Compounds **22** and **23** were obtained with 9.0% and 10.5% yields, respectively.

**Scheme 6:**
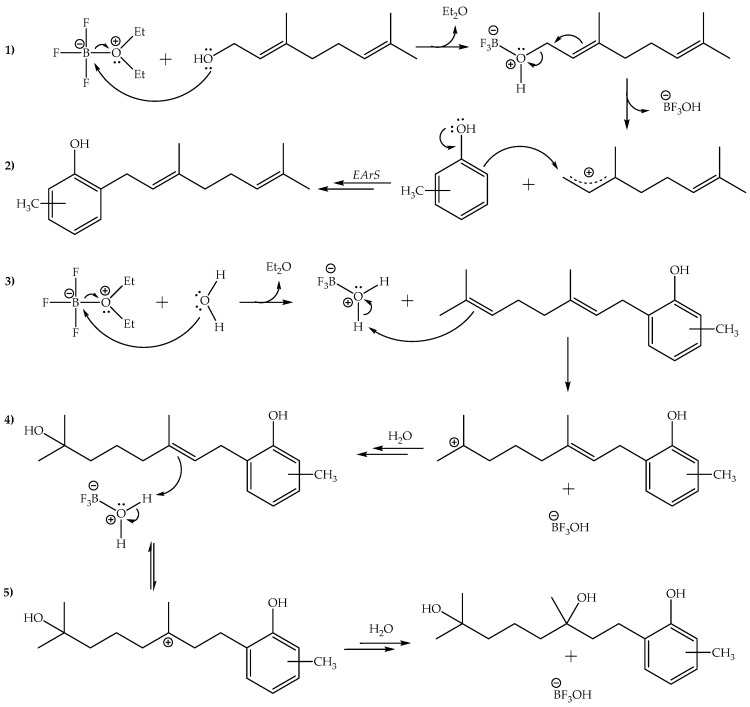
Proposed mechanism for the formation of Compounds **22** and **23**.

The formation of Compounds **22** and **23** may be explained by the proposed mechanism depicted in Scheme 6.

In the first step, an allylic carbocation is formed by the reaction of BF_3_·Et_2_O with geraniol, which is then coupled with phenol via Electrophilic Aromatic Substitution (EArS) (Step 2). In presence of water, the adduct BF_3_·H_2_O is presumably formed by nucleophilic displacement of an ether molecule by H_2_O (Step 3). Subsequently, this adduct reacts with the geranyl chain by a Markovnikov-type addition, forming a stable tertiary carbocation, which is then hydrated by reaction with a water molecule (Steps 3 and 4). Finally, the remaining olefinic bond is hydrated by BF_3_·H_2_O, and a completely hydrated geranyl chain is obtained (Step 5). It is worth mentioning that water is added 24 h after the coupling reaction has been started. This means that Step 3 begins when most of the geranylphenol has already been formed.

**Scheme 7:**
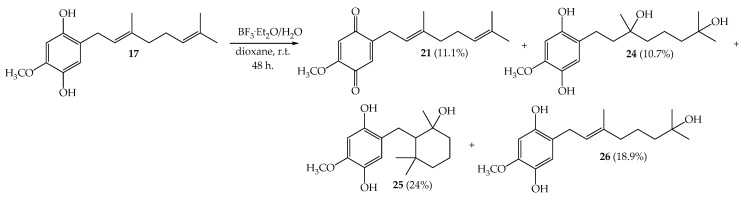
Synthesis of Compounds **21** and **24**–**26**.

Based on this result, our second approach consists of the direct hydration of the side chain by the reaction of a geranylated phenol with a Lewis acid (BF_3_·Et_2_O) in dioxane and in the presence of water. Compound **17** was submitted to this reaction, and compounds **21**, **24**–**26** were obtained with 11.1%, 10.7%, 24% and 18.9% yields, respectively (Scheme 7).

**Scheme 8:**
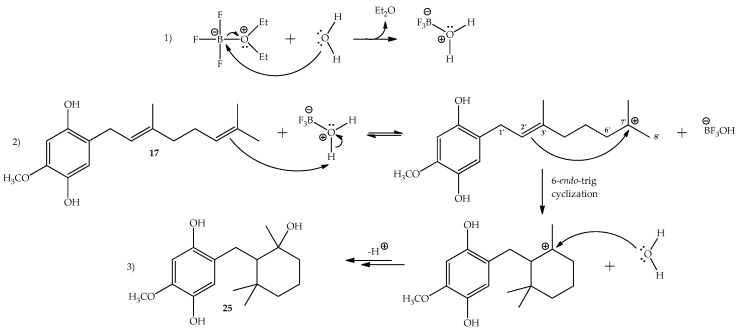
Proposed mechanism for formation of Compound **25**. Reaction of Lewis acid (BF_3_·Et_2_O) with water, addition Markovnikov of H by BF_3_H_2_O adduct on C-6′ position of geranyl chain and carbocation formation in C-7′, 6-endo-trig cyclization and later geranyl chain hydration.

Compounds **24** and **26** may be formed through Steps 3–5 of the mechanism proposed in Scheme 6. However, in this reaction, Compound **25** is formed by cyclization of the tertiary carbocation formed in Step 3, the formation of tertiary carbocation in the C-7′ position of geranyl chain, 6-endo-trig cyclization from the C2′-C3′ double bond and hydration by the subsequent nucleophilic attack of water on the tertiary carbocation in the C-3′ position (Scheme 8). 

The carbocation intermediates appearing in Scheme 6 and Scheme 8 have been proposed for coupling of phenol with geraniol and various reactions of geraniol in acidic aqueous solution [39,40].

Compounds **14**–**18**, **20**, **22**–**26** are new, and their structural characterization is described in the next section.

### 2.2. Structure Determination

**Figure 3:**
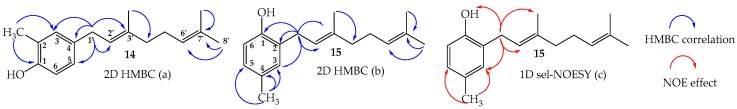
Main observed correlations: 2D Heteronuclear Multiple Bond Correlation (HMBC), Compound **14** (**a**) and Compound **15** (**b**); 1D Nuclear Overhauser Effect Spectroscopy (NOESY) Compound **15** (**c**).

The chemical structures of all compounds synthesized in this work were mainly established by 1D and 2D Nuclear Magnetic Resonance (NMR) spectroscopy techniques. All NMR spectra are given in Figure S1. In this section, the NMR data used to determine the chemical structure of new compounds, geranylated phenol derivatives (**14**–**18**), geranylated quinone (**20**) and hydrated geranylphenols (**22**–**26**), are discussed in detail. Compound **19** has been already reported, but it has been included in this section because the NMR assignation given in literature is not right [41]. Compounds **14**–**18**: The ^1^H-NMR spectrum of Compound **14** shows a pattern characteristic of aromatic tri-substitution, *i.e.*, singlet signal at 6.93 ppm (1H, H-3, meta-coupling of H-3 with H-5 was not detected); doublet at 6.88 ppm (1H, *J* = 8.0, H-5); and doublet at 6.69 ppm (1H, *J* = 8.0, H-6). The position of the geranyl chain on the aromatic ring has been established by two-dimensional (2D) Heteronuclear Multiple Bond Correlation (HMBC) correlations. In this spectrum, a ^2^J_H-C_ coupling of H-1′ with C-4 (δ_C_ = 133.9 ppm) and C-2′ (δ_C_ = 123.6 ppm) and a ^3^J_H-C_ coupling between the signals of C-1, C-3, C-3′ and C-5 at δ_C_ = 130.9, 135.7 and 126.7 ppm, respectively, were observed. Further correlations at ^3^J_H-C_ between the CH_3_-Ar group (δ_H_ = 2.23 ppm) with C-1 and C-3 at δ_C_ = 151.8 and 130.9 ppm, respectively, were observed (Figure 3a). On the other hand, the ^1^H-NMR spectrum of Compound **15** shows a singlet signal at 6.93 ppm (1H, H-3, meta-coupling of H-3 with H-5 was not detected) and two doublet signals at 6.92 ppm (1H, *J* = 8.7 Hz, H-5) and 6.73 (1H, *J* = 8.7 Hz, H-6). Additionally, a signal appearing at 5.07 ppm (s, 1H) was assigned to the OH group. The aromatic substitution pattern shows unequivocally that the geranyl chain is attached to the ortho position at hydroxyl groups. The position of the geranyl chain on the aromatic ring has been confirmed by 2D HMBC correlations. In this spectrum, the signal at δ_H_ = 3.35 ppm assigned to H-1′ (2H d, *J* = 7.2 Hz) shows ^3^J_H-C_ coupling with C-1 (δ_C_ = 152.1), C-3 (δ_C_ = 127.8) and C-3′ (δ_C_ = 138.1 ppm) and ^2^J_H–C_ coupling with C-2 and C-2′ (δ_C_ = 126.6 and 121.8 ppm, respectively; Figure 3b) Additionally, the signal at δ_H_ = 3.35 ppm (H-1′) showed spatial correlations with the signals at δ_H_ = 6.93, 5.07 and 1.79 ppm, assigned to H-3, OH and CH_3_-C3′, respectively; while the signal at δ_H_ = 2.23 ppm (s, CH_3_-Ar) showed spatial correlations with H-3 and H-5 (Figure 3c).

In the ^1^H-NMR spectrum of the acetylated derivative **16**, a singlet at δ_H_ = 2.29 ppm (3H, CH_3_CO) was observed. Additionally, in the ^13^C NMR spectrum, the signals appearing at δ_C_ = 169.6 (C=O) and 20.8 (CH_3_) ppm confirmed the presence of monoacetylated derivative **16**.

**Figure 4:**
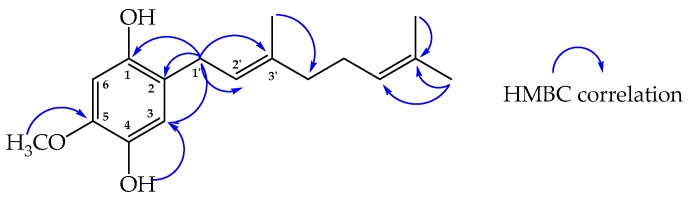
Major 2D HMBC observed correlations for Compound **17**.

Compound **17**: In the ^1^H-NMR spectrum, a pattern characteristic of aromatic tetra-substitution, *i.e.*, two singlet signals at 6.67 (1H, H-3) and 6.43 (1H, H-6), was observed. The position of the geranyl chain on the aromatic ring was established by two-dimensional (2D) HMBC correlations. In this spectrum, a ^2^J_H–C_ coupling of H-1′ with C-2 (δ_C_ = 118.5 ppm) and C-2′ (δ_C_ = 121.8 ppm) and a ^3^J_H–C_ coupling between the signals of C-1, C-3 and C-3′ at δ_C_ = 147.6, 115.3 and 139.2 ppm, respectively, were observed. In addition, a correlation at ^3^J_H–C_ between the CH_3_O group (δ_H_ = 3.83 ppm) and C-5 (δ_C_ = 145.4 ppm) and correlations between ^2^J_H–C_ and ^3^J_H–C_ of the OH-C4 group (δ_H_ = 5.15 ppm) with C-3 (δ_C_ = 115.3 ppm) were also observed (Figure 4).

In the ^1^H-NMR spectrum of the acetylated derivative **18**, two singlet signals at δ_H_ = 2.29 and 2.28 ppm (each 3H, CH_3_CO) were observed. Additionally, in the ^13^C NMR spectrum, the signals appearing at δ = 169.2 (COCH_3_-C4), 168.9 (COCH_3_-C1) ppm and δ = 20.8 (CH_3_COO-C1) and 20.6 (CH_3_COO-C4) ppm, confirmed the presence of diacetylated derivative **18**.

**Figure 5:**
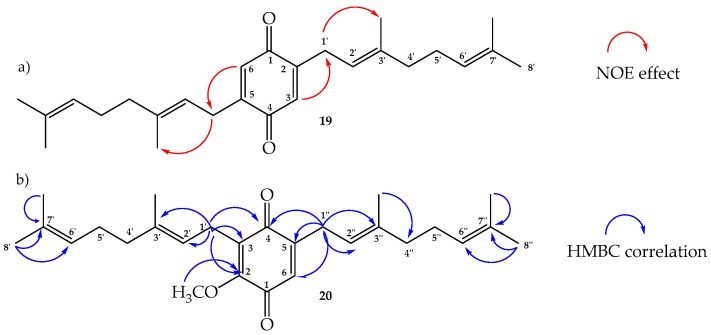
Main observed correlations: 1D NOE Compound **19** (**a**), 2D HMBC Compound **20** (**b**).

Compound **19**: The symmetrical molecular structure was confirmed by the substitution pattern in the olefin zone and by the intensity of integrated signals of hydrogen atoms in quinone and olefinic portion. For instance, the signal at δ_H_ = 6.70 ppm (s, 2H, H-3 and H-6) indicates the presence of two identical H. In a previous report, two different signals were found and assigned to these H (δ_H_ = 6.48 ppm, 1H, s, H-13 and δ_H_ = 6.52 ppm, 1H, s, H-16). A detailed assignment of ^1^H-NMR signals is given in the experimental part, and the corresponding spectrum is shown in the Supplementary Material. Additionally, spatial correlations (NOE) were observed for the signals at δ_H_ = 6.70 ppm and at δ_H_ = 3.21 ppm (4H, d, *J* = 6.8, H-1′) and for the latter and the signal at δ_H_ = 1.73 ppm, assigned to CH_3_-C3′ (Figure 5a). Finally, in the ^13^C NMR spectrum, only one signal at δ_C_ = 187.6 ppm (C-1 and C-4) of the carbonyl group was observed, confirming the symmetrical structure of Compound **19**.

Compound **20**: The presence of double geranyl chain substitution on the quinone nucleus was confirmed by the observation of two doublet signals in the ^1^H-NMR spectrum at δ_H_ = 3.15 ppm (2H, *J* = 7.4 Hz) and 3.12 ppm (2H, *J* = 7.0 Hz), which were assigned to the hydrogens H-1′ and H-2′′, respectively. Additionally, the presence of only one hydrogen at δ_H_ = 6.32 ppm (1H, s, H-6) demonstrates the degree of tetra-substitution on the quinone moiety. Differentiation between geranyl chains was established by the HMBC correlations observed for H-1′ at ^3^J_H–C_ with C-4 (δ_C_ = 187.9 ppm; C=O) and H-1′ at ^3^J_H–C_ with C-2 (δ_C_ = 155.1 ppm; C-OCH_3_) and at ^2^J_H–C_ with C-3 (δ_C_ = 131.9 ppm) (Figure 5b). Similarly, the signal of H-1′′ showed ^3^J_H–C_ correlations with C-4 (δ_C_ = 187.9 ppm; C=O) and C-6 (δ_C_ = 130.5 ppm) and ^2^J_H–C_ with C-5 (δ_C_ = 148.3 ppm) (Figure 5b).

**Figure 6:**
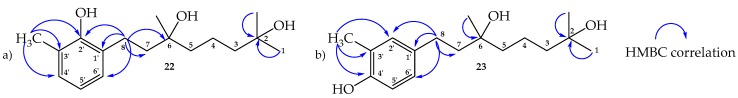
Main 2D HMBC observed correlations for: (**a**) Compound **22**; (**b**) Compound **23**.

Compound **22**: The ^1^H-NMR spectrum shows a pattern characteristic of aromatic tri-substitution, *i.e.*, doublet signals at δ_H_ = 6.95 ppm (*J* = 7.3 Hz, 1H, H-4′) and δ_H_ = 6.90 (*J* = 7.4 Hz, 1H, H-6′), a double doublet signal at δ_H_ = 6.70 (*J* = 7.3 and 7.4 Hz, 1H, H-5′). The position of the geranyl chain on the aromatic ring was established by two-dimensional (2D) HMBC correlations. In this spectrum, a ^2^J_H-C_ coupling of H-8 (δ_H_ = 2.78–2.74, m, 2H) with C-1′ (δ_C_ = 120.4 ppm) and a ^3^J_H-C_ coupling between the signals of C-2′and C-6′ at δ_C_ = 151.9 and 126.9 ppm, respectively, were observed. In addition, correlations at ^3^J_H-C_ between the CH_3_-Ar group (δ_H_ = 2.16 ppm) with C-2′ and C-4′ at δ_C_ = 151.9 and 128.4 ppm, respectively, and a ^2^J_H-C_ with C-3′ (δ_C_ = 126.2) were observed (Figure 6a). The presence of two hydroxyl groups in the geranyl chain was confirmed by the observation of two tertiary carbinolic signals at δ_C_ = 75.8 and 71.0 ppm in the ^13^C NMR spectrum. These were assigned to carbons C-6 and C-2, respectively, by two-dimensional (2D) HMBC correlations. Thus, H-8 showed correlation at ^3^J_H-C_ with carbinolic carbon at C-6 (δ_C_ = 75.8 ppm), whereas the methyl groups at δ_C_ = 29.2 ppm (CH_3_-1 and CH_3_-C2) showed correlations at ^2^J_H-C_ with C-2 (δ_C_ = 71.0 ppm) (Figure 6a).

Compound **23**: A similar analysis was conducted to elucidate the structure of this compound. The ^1^H-NMR spectrum shows a pattern characteristic of aromatic tri-substitution, *i.e.*, a singlet signal at δ_H_ = 6.95 ppm (1H, H-2′, meta-coupling of H-2′ with H-6′ was not detected), a doublet signal at δ_H_ = 6.90 (*J* = 8.1 Hz, 1H, H-6′) and a doublet signal at δ_H_ = 6.68 (*J* = 8.1 and 1H, H-5′). The position of the geranyl chain on the aromatic ring was established by two-dimensional (2D) HMBC correlations. In this spectrum, a ^2^J_H-C_ coupling of H-8 (δ_H_ = 2.65–2.52, m, 2H) with C-1′ (δ_C_ = 135.5 ppm) and a ^3^J_H-C_ coupling between the signals of C-2′and C-6′ at δ_C_ = 130.9 and 126.7 ppm, respectively, were observed. In addition, correlations at ^3^J_H-C_ between the CH_3_-Ar group (δ_H_ = 2.22 ppm) with C-2′ and C-4′ at δ_C_ = 130.9 and 151.6 ppm, respectively, and a ^2^J_H-C_ with C-3′ (δ_C_ = 123.4) were observed (Figure 6b). The presence of two hydroxyl groups in the geranyl chain was confirmed by the observation of two tertiary carbinolic signals at δ_C_ = 72.9 and 71.3 ppm in the ^13^C NMR spectrum. These signals were assigned to carbons C-6 and C-2, respectively, by two-dimensional (2D) HMBC correlations. Thus, the CH_3_-C6 group showed correlation at ^2^J_H-C_ with carbinolic carbon at C-6 (δ_C_ = 72.9 ppm), while the methyl groups at δ_C_ = 31.2 and 29.9 ppm (CH_3_-1 and CH_3_-C2, respectively) showed correlations at ^2^J_H-C_ with C-2 (δ_C_ = 71.3 ppm) (Figure 6b).

**Figure 7:**
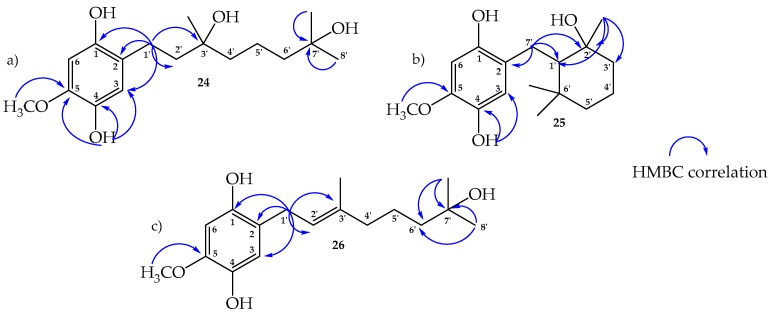
Major 2D HMBC observed correlations for Compounds **24** (**a**), **25** (**b**) and **26** (**c**).

Because Compound **24** was obtained from **17** by the hydration reaction of it, the aromatic substitution pattern was maintained for Compounds **24** and **25**. Thus, in Compound **24**, the presence of two hydroxyl groups in the geranyl chain was confirmed by the observation of two tertiary carbinolic signals at δ_C_ = 75.8 and 70.9 ppm in the ^13^C NMR spectrum. These signals were assigned to carbons C-3′ and C-7′, respectively, by two-dimensional (2D) HMBC correlations. Thus, the CH_3_-C7′ and CH_3_-8′ groups showed correlation at ^2^J_H-C_ with carbinolic carbon at C-7′ (δ_C_ = 70.9 ppm), and therefore, the signal at δ_C_ = 75.8 ppm was unequivocally assigned to C-3′ (Figure 7a); while for Compound **25**, the methylene group (at δ_C_ = 22.7 ppm, assigned as C-7′) showed a correlation at ^2^J_H-C_ with C-2 (δ_C_ = 114.2 ppm) and C-1′ (δ_C_ = 48.3 ppm) and ^3^J_H-C_ with a tertiary carbinolic carbon at δ_C_ = 76.7 ppm assigned to C-2′. Additionally, the CH_3_-C2′ group at δ_H_ = 1.20 ppm (3H, s) showed coupling at ^2^J_H-C_ with C-2′ and ^3^J_H-C_ with tertiary C-1′ (δ_C_ = 48.3 ppm) (Figure 7b) Thus, the cyclohexane structure is confirmed for geranyl chain. Finally, mono-hydroxylation in the geranyl chain for Compound **26** was mainly established by ^13^C NMR data and 2D HMBC correlations. Only one signal of carbinolic carbon at δ_C_ = 70.9 ppm in the ^13^C NMR spectrum was observed, and the methyl groups at δ_H_ = 1.22 (6H, s, CH_3_-C7′ and H-8′) showed ^2^J_H-C_ correlations with this carbon (C-7′, δ_C_ = 70.9 ppm) (Figure 7c). In addition, these methyl groups showed ^3^J_H-C_ correlations with C-6′ (δ_C_ = 43.3 ppm) (Figure 7c).

### 2.3. In Vitro Antifungal Activity against B. cinerea.

**Figure 8:**
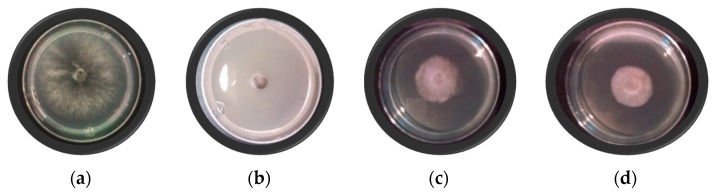
Effect of hydrated geranylphenol **26** on the *in vitro* mycelial growth of *B. cinerea*. (**a**) Negative control; the medium contains only Potato Dextrose Agar (PDA) and 1% ethanol. (**b**) Positive control; Captan at 250 ppm. (**c**) Compound **26** at 150 ppm. (**d**) Compound **26** at 250 ppm.

All studied compounds (**14**–**26**) were tested for *in vitro* antifungal activity on the mycelial growth of *B. cinerea* strain GM7 using the agar radial assay with Potato Dextrose Agar (PDA). Figure 8 shows an assay where the *B. cinerea* mycelium grows in medium containing only PDA and 1% ethanol (Figure 8a, negative control), Captan at 250 ppm (Figure 8b, used in this study as a positive control) and two different concentrations of Compound **26** (Figure 8c, 150 ppm; Figure 8d, 250 ppm).

The inhibition of mycelial growth is evaluated by measuring colony diameters in the presence and absence of the tested compounds. The results, expressed as the percentage of inhibition, are summarized in Table 1.

The data indicate that geranylated derivatives of *o*- and *p*-cresol (**14**–**16**) have no effect on the mycelial growth of *B. cinerea*. However, the methoxy derivatives of geranylated *p*-cresol (**17** and **18**) exhibit a significative increase in the inhibitory activity. This result is in line with previous work where a family of methoxy geranylated derivatives was studied [30].

On the other hand, geranylated quinones (**19**–**21**) show an important activity (greater than 50% at the higher tested concentrations) that is independent of the number of geranyl chains. In the case of geranylated phenols, it was found that antifungal activity decreases with the increasing number of prenyl chains [29,30].

All hydrated geranylphenols exhibit activities on mycelial growth inhibition that are in the range 30%–95% at 250 ppm. A comparison of the percentages of inhibition measured for geranylated phenol and their respective hydrated compound, *i.e.*, **14** with **23**, **17** with **24** and **26**, shows that the latter are more active than the parent compound. This effect is larger for compounds carrying only one hydroxyl group in the side chain (**25** and **26**) than for completely hydrated compounds (**22**–**24**). In other words, incorporation of hydroxyl groups in the side chain, by hydration of the geranyl chain, brings about an enhancing effect on the antifungal activity. Previous work was focused on the effect of substitution in the phenol ring, and the results suggested that the inhibition effect depends mainly on the presence of the prenyl chain [29,31]. In this context, these results are important because, as far as we know, this is the first report of the effect of the side chain structure on the fungicide activity of geranylated compounds.

Finally, it is interesting to stress that Compound **26** stands out as being as active as Captan, a fungicide that is currently used for infection control in some crops.

## 3. Experimental Section

### 3.1. General

Chemicals were obtained from Merck (Darmstadt, Germany) or Aldrich (St. Louis, MO, USA) and were used without further purification. A detailed description of conditions used to register Fourier transform infrared (FT-IR) spectra, high resolution mass spectra and ^1^H, ^13^C, ^13^C DEPT-135, selective gradients 1D ^1^H NOESY, gs-2D Heteronuclear Single Quantum Coherence (HSQC) and gs-2D HMBC spectra has been given elsewhere [30]. Silica gel (Merck 200–300 mesh) was used for Column Chromatography (C.C.) and silica gel plates HF_254_ for thin layer chromatography (TLC). TLC spots were detected by heating after spraying with 25% H_2_SO_4_ in H_2_O.

### 3.2. Synthesis

#### 3.2.1. Coupling Reaction in Presence of Nitrogen

The coupling of geraniol and phenols was carried out using boric trifluoride etherate BF_3_·Et_2_O as the catalyst and dioxane as the solvent. Experimental details for a typical reaction have been given elsewhere [30].

(*E*)-4-(3,7-dimethylocta-2,6-dienyl)-2-methylphenol (**14**):

Coupling of *o*-cresol (1.0 g, 9.3 mmol) and geraniol (1.5 g, 9.7 mmol) was carried out in dioxane (20 mL) with BF_3_·Et_2_O (0.9 mL, 7.2 mmol) as the catalyst. Two fractions were obtained from the C.C. Fraction I: Compound **14** (64 mg, 3.1% yield) obtained as a yellow viscous oil; Fraction II: unreacted *o*-cresol (922 mg) that was recovered. Compound **14**: IR (cm^−1^) 3385, 2966, 2923, 2854, 1668, 1611, 1508, 1453, 1377, 1262, 1205, 1115, 772; ^1^H-NMR (CDCl_3_, 400.1 MHz) δ 6.93 (1H, s, H-3), 6.88 (1H, d, *J* = 8.0, H-5), 6.69 (1H, d, *J* = 8.0, H-6), 5.32–5.29 (1H, m, H-2′), 5.11–5.09 (1H, m, H-6′), 4.55 (1H, s, OH), 3.25 (2H, d, *J* = 7.2, H-1′), 2.23 (3H, s, CH_3_-C2), 2.12–2.08 (2H, m, H-5′), 2.06–2.04 (2H, m, H-4′), 1.69 (3H, s, CH_3_-C3′), 1.68 (3H, s, H-8′), 1.60 (3H, s, CH_3_-C7′); ^13^C NMR (CDCl_3_, 100.6 MHz) δ 151.8 (C-1), 135.7 (C-3′), 133.9 (C-4), 131.4 (C-7′), 130.9 (C-3), 126.7 (C-5), 124.3 (C-6′), 123.6 (C-2′), 123.4 (C-2), 114.8 (C-6), 39.7 (C-4′), 33.3 (C-1′), 26.6 (C-5′), 25.7 (C-8′), 17.7 (CH_3_-C7′), 16.1 (CH_3_-C3′); 15.8 (CH_3-_C4); MS *m*/*z* (%) M^+^ 244 (48.3), 201 (16.7), 175 (100), 160 (36.7), 147 (31.7), 133 (35.0), 121 (68.3), 106 (13.3), 91 (20.0), 69 (28.3), 41 (31.7).

(*E*)-2-(3,7-dimethylocta-2,6-dienyl)-4-methylphenol (**15**):

Coupling of *p*-cresol (1.02 g, 9.4 mmol) and geraniol (1.46 g, 9.4 mmol) was carried out in dioxane (20 mL) with BF_3_·Et_2_O (1.17 mL, 9.5 mmol) as the catalyst. Two fractions were obtained from the C.C. Fraction I: Compound **15** (264 mg, 12% yield) obtained as a yellow viscous oil; Fraction II: unreacted *p*-cresol (728 mg) that was recovered. Compound **15**: IR (cm^−1^) 3446, 2966, 2919, 2857, 1611, 1506, 1446, 1376, 1260, 1197, 1105, 1040, 924, 810; ^1^H-NMR (CDCl_3_, 400.1 MHz) δ 6.93 (1H, s, H-3), 6.92 (1H, d, *J* = 8.7, H-5), 6.73 (1H, d, *J* = 8.7, H-6), 5.36–5.32 (1H, m, H-2′), 5.12–5.09 (1H, m, H-6′), 5.07 (1H, s, OH), 3.35 (2H, d, *J* = 7.2, H-1′), 2.28 (3H, s, CH_3_-C4), 2.16–2.14 (2H, m, H-5′), 2.11–2.09 (2H, m, H-4′), 1.79 (3H, s, CH_3_-C3′), 1.71 (3H, s, CH_3_-C7′), 1.62 (3H, s, H-8′); ^13^C NMR (CDCl_3_, 100.6 MHz) δ 152.1 (C-1), 138.1 (C-3′), 131.9 (C-7′), 130.5 (C-5), 129.8 (C-4), 127.8 (C-3), 126.6 (C-2), 123.8 (C-7′), 121.8 (C-2′), 115.6 (C-6), 39.7 (C-4′), 29.7 (C-1′), 26.4 (C-5′), 25.7 (C-8′), 20.5 (CH_3-_C4); 17.7 (CH_3_-C7′), 16.0 (CH_3_-C3′); MS *m*/*z* (%) M^+^ 244 (70), 201 (33.3), 175 (88.3), 159 (65), 147 (40), 133 (35), 121 (100: M^+^-123 (C_9_H_15_)), 105 (30), 91 (36.7), 69 (40), 41 (45).

(*E*)-2-(3,7-dimethylocta-2,6-dienyl)-5-methoxybenzene-1,4-diol (**17**):

Coupling of 2-methoxyhydroquinone (2.02 g, 14.4 mmol) and geraniol (2.36 mL, 13.2 mmol) was carried out in dioxane (20 mL) with BF_3_·Et_2_O (1.62 mL, 12.9 mmol) as the catalyst. Two fractions were obtained from the C.C. Fraction I: Compound **17** (233 mg, 5.9% yield) was obtained as a brown viscous oil; Fraction II: unreacted 2-methoxyhydroquinone (1.76 g) that was recovered. Compound **17**: IR (cm^−1^) 3420, 2965, 2924, 2852, 1603, 1520, 1446, 1196, 1105, 835; ^1^H-NMR (CDCl_3_, 400.1 MHz) δ 6.67 (1H, s, H-3), 6.43 (1H, s, H-6), 5.28 (1H, t, *J* = 7.2 Hz, H-2′), 5,15 (1H, bs, OH-C4), 5.06 (1H, t, *J* = 5.5 Hz, H-6′), 4.87 (1H, bs, OH-C1), 3.83 (3H, s, CH_3_O), 3.26 (2H, d, *J* = 7.2 Hz, H-1′), 2.12–2.10 (2H, m, H-5′), 2.08–2.05 (2H, m, H-4′), 1.76 (3H, s, CH_3_-C3′), 1.69 (3H, s, H-8′), 1.60 (3H, s, CH_3_-C7′); ^13^C NMR (CDCl_3_, 100.6 MHz) δ 147.6 (C-1), 145.4 (C-5), 139.2 (C-3′), 138.6 (C-4), 132.1 (C-7′), 123.8 (C-6′), 121.8 (C-2′), 118.5 (C-2), 115.3 (C-3), 100.4 (C-6), 56.1 (CH_3_O-C5), 39.7 (C-4′), 29.4 (C-1′), 26.4 (C-5′), 25.7 (C-8′), 17.7 (CH_3_-C7′), 16.2 (CH_3_-C3′); MS *m*/*z* (%) 276 (39.5: M^+^), 191 (21), 175 (9.9), 153 (100: M^+^-123 (C_9_H_15_)), 91 (4.9), 69 (16), 41 (16).

#### 3.2.2. Coupling Reaction in the Absence of Nitrogen and with Added Water

The main difference in the experimental procedure of this reaction is that, after the addition and stirring were completed, 5 mL of H_2_O were added, and the stirring was continued for another 24 h. The crude was chromatographed on silica gel with petroleum ether/EtOAc mixtures of increasing polarity (19.8:0.2 → 8.0:12.0).

2-Geranylhydroquinone (**1**), 2-geranylquinone (**3**) and 2,5-bisgeranylquinone (**19**):

Coupling of 1,4-hydroquinone (1.01 g, 9.2 mmol) and geraniol (1.35 g, 5.5 mmol) was carried out in dioxane (30 mL) with BF_3_·Et_2_O (0.46 g, 3.2 mmol) as the catalyst. Three fractions were obtained from the C.C. Fraction I: Compound **19** (42 mg, 1.9% yield) obtained as a brown viscous oil. Compound **19**: ^1^H-NMR (CDCl_3_, 400.1 MHz) δ 6.70 (2H, s, H-3 and H-6), 5.03 (2H, t, *J* = 6.8 Hz, H-2′), 4.94 (2H, t, *J* = 6.3 Hz, H-6′), 3.21 (4H, d, *J* = 6.8, H-1′), 2.07–2.02 (4H, m, H-5′), 1.98–1.95 (4H, m, H-4′), 1.73 (6H, s, CH_3_-C3′), 1.68 (6H, s, H-8′), 1.57 (6H, s, CH_3_-C7′); ^13^C NMR (CDCl_3_, 100.6 MHz) δ 187.6 (C-1 and C-4), 143.6 (C-2 and C-5), 137.5 (C-3′ and C-3′′), 136.2 (C-3 and C-6), 131.5 (C-7′ and C-7′′), 123.9 (C-6′ and C-6′′), 119.5 (C-2′ and C-2′′), 39.7 (C-4′ and C-4′′), 26.5 (C-1′ and C-1′′), 25.7 (C-5′ and C-5′′), 25.3 (C-8′ and C-8′′), 17.7 (CH_3_-C7′ and CH_3_-C7′′), 16.4 (CH_3_-C3′ and CH_3_-C3′′). Fraction II: Compound **3** (166 mg, 7.6% yield) obtained as a brown viscous oil. Fraction III: Compound **1** (616 mg, 28% yield) obtained as a brown viscous oil. The spectroscopic data (IR, MS and NMR) for **1** and **3** were consistent with those previously reported [26].

3,5-Bis((*E*)-3,7-dimethylocta-2,6-dienyl)-2-methoxycyclohexa-2,5-diene-1,4-dione (**20**):

Coupling of 2-methoxyhydroquinone (500 mg, 3.6 mmol) and geraniol (0.65 mL, 3.6 mmol) was carried out in dioxane (20 mL) with BF_3_·Et_2_O (0.46 g, 3.2 mmol) as the catalyst. Two fractions were obtained from the C.C. Fraction I: Compound **20** (60 mg, 4.1% yield) was obtained as a brown viscous oil; Fraction II: unreacted 2-methoxyhydroquinone (419 mg) that was recovered. Compound **20**: IR (cm^−1^) 2966, 2924, 2854, 1649, 1602, 1446, 1376, 1323, 1207, 1161, 1121, 954, 887; ^1^H-NMR (CDCl_3_, 400.1 MHz) δ 6.32 (1H, s, H-6), 5.15–5.13 (1H, m, H-2′), 5.11–5.08 (1H, m, H-6′), 5.07–5.04 (1H, m, H-2′′), 5.03–5.01 (1H, m, H-6′′), 3.99 (3H, s, CH_3_O-C2), 3.15 (2H, d, *J* = 7.4 Hz, H-1′), 3.12 (2H, d, *J* = 7.0 Hz, H-1′′), 2.11–2.09* (2H, m, H-5′), 2.07–2.05 (2H, m, H-4′′), 2.04–2.02* (2H, m, H-5′′), 1.97–1.95 (2H, m, H-4′), 1.73 (3H, s, CH_3_-C3′′), 1.69** (3H, s, H-8′), 1.67** (3H, s, H-8′′), 1.64*** (3H, s, CH_3_-C7′), 1.63 (3H, s, CH_3_-C3′), 1.60*** (3H, s, CH_3_-C7′′); ^13^C NMR (CDCl_3_, 100.6 MHz) δ 187.9 (C-4), 184.3 (C-1), 155.1 (C-2), 148.3 (C-5), 139.7 (C-3′′), 136.9 (C-3′), 131.9 (C-3), 131.8* (C-7′), 131.4* (C-7′′), 130.5 (C-6), 124.1 (C-6′′), 123.9 (C-6′), 120.1 (C-2′), 118.0 (C-2′′), 60.9 (CH_3_O-C2), 39.7 (C4′), 39.6 (C4′′), 27.3 (C-1′′), 26.6** (C-5′), 26.4** (C-5′′), 25.7*** (C-8′), 25.6*** (C-8′′), 22.5 (C-1′), 17.7^¤^ (CH_3_-C-7′), 17.6^¤^ (CH_3_-C-7′′), 16.1^¤¤^ (CH_3_-C-3′), 16.1^¤¤^ (CH_3_-C-3′′); MS *m*/*z* (%)M^+^ 410 (8.8), 327 (100: M^+^-83 (C_6_H_11_)), 243 (3.5), 227 (8.8), 207 (3.5), 189 (5.3), 91 (3.5), 69 (12.3), 41 (10.5). * ** *** ^¤^^¤¤^: interchangeable signals.

8-(2-Hydroxy-3-methylphenyl)-2,6-dimethyloctane-2,6-diol (**22**) and 8-(4-hydroxy-3-methylphenyl)- 2,6-dimethyloctane-2,6-diol (**23**):

Coupling of *o*-cresol (1.0 g, 9.3 mmol) and geraniol (1.55 g; 10.0 mmol) was carried out in dioxane (20 mL) with BF_3_·Et_2_O (1.2 mL, 10.0 mmol) as the catalyst. Three fractions were obtained from the C.C. Fraction I: unreacted *o*-cresol (473 mg) that was recovered. Fraction II: Compound **22** (225 mg, 9.0% yield) obtained as a brown viscous oil. IR (cm^−1^) 3375, 2968, 2866, 1595, 1467, 1376, 1264, 1220, 1152, 1112, 935, 763; ^1^H-NMR (CDCl_3_, 400.1 MHz) δ 6.95 (1H, d, *J* = 7,3 Hz, H-4′), 6.90 (1H, d, *J* = 7,4 Hz, H-6′), 6.70 (1H, dd, *J* = 7,4 and 7.3 Hz, H-5′), 2.78–2.74 (2H, m, H-8), 2.16 (3H, s, CH_3_-C3′), 1.85–1.73 (2H, m, H-7), 1.68–1.62 (2H, m, H-5), 1.56–1.52 (2H, m, H-4), 1.51–1.47 (2H, m, H-3), 1.29 (3H, s, CH_3_-C6), 1.23 (6H, s, H-1 and CH_3_-C2); ^13^C NMR (CDCl_3_, 100.6 MHz) δ 151.9 (C-2′), 128.2 (C-4′), 126.9 (C-6′), 126.2 (C-3′), 120.4 (C-1′), 118.8 (C-5′), 75.8 (C-6), 71.0 (C-2), 44.3 (C-3), 40.5 (C-5), 31.3 (C-7), 29.3 and 29.2 (C-1 and CH_3_-C2), 24.2 (CH_3_-C6), 22.3 (C-8), 18.4 (C-4), 16.0 (CH_3_-C3**′**). MS *m*/*z* (%) M^+^ 280 (< 1%), 262 (55.4), 244 (12.5), 229 (14.3), 201 (19.6), 188 (10.7), 173 (64.3), 161 (48.2), 145 (10.7), 121 (100: M^+^-159 (C_9_H_19_O_2_)); 109 (16.1), 91 (28.6), 77 (12.5), 59 (14.3), 43 (14.3). Fraction III: Compound **23** (263 mg, 10.5% yield) obtained as a brown viscous oil. IR (cm^−1^) 3358, 2970, 2933, 2866, 1509, 1457, 1376, 1263, 1222, 1117, 1001, 980, 816; ^1^H-NMR (CDCl_3_, 400.1 MHz) δ 6.95 (1H, s, H-2′), 6.90 (1H, d, *J* = 8,1 Hz, H-6′), 6.68 (1H, d, *J* = 8,1 Hz, H-5′), 2.65–2.52 (2H, m, H-8), 2.22 (3H, s, CH_3_-C3′), 1.83–1.73 (1H, m, H-7), 1.71–1.65 (2H, m, H-3), 1.64–1.61 (1H, m, H-7), 1.55–1.49 (1H, m, H-5), 1.48–1.42 (2H, m, H-4), 1.41–1.36 (1H, m, H-5), 1.24 (9H, s, CH_3_-C6, CH_3_-C2 and H-1); ^13^C NMR (CDCl_3_, 100.6 MHz) δ 151.6 (C-4′), 135.5 (C-1′), 130.9 (C-2′), 126.7 (C-6′), 123.4 (C-3′), 114.7 (C-5′), 72.9 (C-6), 71.3 (C-2), 45.7 (C-7), 36.9 (C-4), 34.8 (C-5), 31.2 (C-1), 29.9 (CH_3_-C2), 29.3 (C-8), 27.7 (CH_3-_C6), 16.5 (C-3), 15.7 (CH_3_-C3′); MS *m*/*z* (%) 281 (2.0: M + 1), 262 (23.5), 244 (41.2), 229 (11.8), 201 (15.7), 187 (15.7), 173 (37.3), 161 (39.2), 121 (100: M^+^-159 (C_9_H_19_O_2_)), 109 (64.7), 91 (15.7), 69 (19.6), 43 (19.6).

#### 3.2.3. Acetylation of Geranylated Phenols

Geranylated phenols were acetylated by following a described acetylation method [30].

(*E*)-2-(3,7-dimethylocta-2,6-dienyl)-4-methylphenyl acetate (**16**):

Acetylation of Compound **15** (100 mg, 0.4 mmol) with Ac_2_O (0.54 g, 5.3 mmol), DMAP (2.0 mg) and pyridine (1.0 mL) in dichloromethane (20 mL) gives Compound **16** as a viscous yellow oil (111.2 mg, 94.8% yield). Compound **16**: IR (cm^−1^) 2966, 2923, 1763, 1447, 1496, 1367, 1213, 1191, 1105, 1010, 901, 824; ^1^H-NMR (CDCl_3_, 400.1 MHz) δ 7.03 (1H, s, H-3), 7.02 (1H, d, *J* = 7.9 Hz, H-5), 6.90 (1H, d, *J* = 7.9 Hz, H-6), 5.26–5.22 (1H, m, H-2′), 5.13–5.09 (1H, m, H-6′), 3.22 (2H, d, *J* = 7.2 Hz, H-1′), 2.32 (3H, s, CH_3_-C4), 2.29 (3H, s, CH_3_CO), 2.13–2.09 (2H, m, H-5′), 2.07–2.02 (2H, m, H-4′), 1.70 (3H, s, CH_3_-C3′), 1.69 (3H, s, H-8′), 1.61 (3H, s, CH_3_-C7′); ^13^C NMR (CDCl_3_, 100.6 MHz) δ 169.6 (CO), 146.6 (C-1), 136.5 (C-3′), 135.6 (C-4), 132.9 (C-2), 131.4 (C-7′), 130.6 (C-3), 127.5 (C-5), 124.1 (C-6′), 121.7 (C-2′), 121.6 (C-6), 39.6 (C-4′), 28.6 (C-1′), 26.5 (C-5′), 25.6 (C-8′), 20.9 and 20.8 (CH_3-_C4 and CH_3_CO), 17.7 (CH_3_-C7′), 16.1 (CH_3_-C3′). MS *m*/*z* (%) M^+^ 286 (8.9), 243 (42.9), 201 (21.4), 187 (10.4), 175 (100: M^+^-111 (C_7_H_11_O)), 159 (53.6), 123 (69.6), 91 (17.9), 69 (28.6), 43 (26.8).

(*E*)-2-(3,7-dimethylocta-2,6-dienyl)-5-methoxy-1,4-phenylene diacetate (**18**):

Reaction of Compound **17** (65 mg, 0.18 mmol) with Ac_2_O (0.54 g, 5.3 mmol), DMAP (2.0 mg) and pyridine (1.0 mL) in dichloromethane (20 mL) gives Compound **18** as a viscous yellow oil (83 mg, 98% yield). Compound **18**: IR (cm^−1^) 2965, 2919, 2854, 1766, 1509, 1445, 1368, 1201, 1181, 1157, 1012, 906; ^1^H-NMR (CDCl_3_, 400.1 MHz) δ 6.87 (1H, s, H-3), 6.65 (1H, s, H-6), 5.20 (1H, t, *J* = 7.2 Hz, H-2′), 5.09 (1H, t, *J* = 5.5 Hz, H-6′), 3.78 (3H, s, CH_3_O), 3.15 (2H, d, *J* = 7.16, H-1′), 2.29 (3H, s, COCH_3_), 2.28 (3H, s, COCH_3_), 2.10–2.07 (2H, m, H-5′), 2.05–2.02 (2H, m, H-4′), 1.68 (3H, s, CH_3_-C3′), 1.65 (3H, s, H-8′), 1.60 (3H, s, CH_3_-C7′), ^13^C NMR (CDCl_3_, 100.6 MHz) δ 169.2 (COCH_3_-C4), 168.9 (COCH_3_-C1), 149.5 (C-5), 146.6 (C-1), 137.4 (C-4), 137.1 (C-3′), 131.5 (C-7′), 125.5 (C-2), 124.1 (C-6′), 123.3 (C-3), 121.1 (C-2′), 106.9 (C-6), 56.1 (CH_3_O), 39.6 (C-4′), 27.7 (C-1′), 26.4 (C-5′), 25.6 (C-8′), 20.8 (CH_3_COO-C1), 20.6 (CH_3_COO-C4), 17.7 (CH_3_-C7′), 16.1 (CH_3_-C3′); MS *m*/*z* (%) M^+^ 360 (22.2), 318 (16.7), 276 (76.7), 207 (22.2), 191 (44.4), 175 (16.7), 153 (100: M^+^-207 (C_13_H_19_O_2_)), 123 (21.0), 91 (4.9), 69 (24.4), 43 (33.3).

#### 3.2.4. Reaction of Geranylated Phenols with BF_3_·Et_2_O

(*E*)-2-(3,7-dimethylocta-2,6-dien-1-yl)-5-methoxycyclohexa-2,5-diene-1,4-dione (**21**), 2-(3,7-dihydroxy-3,7-dimethyloctyl)-5-methoxybenzene-1,4-diol (**24**), 2-((2-hydroxy-2,6,6-trimethylcyclohexyl)methyl)-5-methoxybenzene-1,4-diol (**25**) and (*E*)-2-(7-hydroxy-3,7-dimethyloct-2-en-1-yl)-5-methoxybenzene-1,4-diol (**26**):

To a solution of Compound **17** (100 mg; 0.36 mmol) in dioxane (30 mL) was slowly added dropwise BF_3_·Et_2_O (0.5 mL, 4.2 mmol) and H_2_O (0.5 mL, 27.8 mmol) with stirring at room temperature and without a N_2_ atmosphere. The crude of the reaction was washed, extracted and chromatographed on silica gel [30]. Five fractions were obtained. Fraction I: unreacted Compound **17** (23 mg) that was recovered. Fraction II: Compound **24** (11 mg, 11.1% yield) obtained as a brown viscous oil. IR (cm^−1^) 2927, 2853, 1674, 1648, 1603, 1454, 1375, 1207, 1174, 987; ^1^H-NMR (CDCl_3_, 400.1 MHz) δ 6.46 (1H, s, H-3), 5.94 (1H, s, H-6), 5.17–5.13 (1H, m, H-2′), 5.09–5.06 (1H, m, H-6′), 3.82 (3H, s, CH_3_O-C5), 3.14 (2H, d, *J* = 7.0 Hz, H-1′), 2.12–2.08 (2H, m, H-4′), 2.07–2.04 (2H, m, H-5′), 1.69 (3H, s, H-8′), 1.61 (3H, s, CH_3_-C3′), 1.60 (3H, s, CH_3_-C7′); ^13^C NMR (CDCl_3_, 100.6 MHz) δ 187.6 (C-1), 182.4 (C-4), 158.7 (C-5), 149.6 (C-2), 140.1 (C-3′), 131.9 (C-7′), 130.3 (C-3), 123.9 (C-6′), 117.8 (C-2′), 107.7 (C-6), 56.2 (CH_3_O-C5), 39.6 (C-5′), 27.3 (C-1′), 26.4 (C-4′), 25.7 (C-8′), 17.7 (CH_3_-C7′), 16.1 (CH_3_-C3′); MS *m*/*z* (%) 274 (8.5: M^+^), 259 (5.1), 191 (100: M^+^-83 (C_6_H_11_)), 176 (10.2), 148 (5.1), 91 (3.4), 69 (5.1), 41 (5.1). Fraction III: Compound **25** (25 mg, 24% yield) obtained as a brown viscous oil. IR (cm^−1^) 355, 3455, 2959, 2927, 2854, 1629, 1509, 1446, 1376, 1278, 1198, 1154, 1121, 1042, 951, 864; ^1^H-NMR (CDCl_3_, 400.1 MHz) δ 6.61 (1H, s, H-3), 6.33 (1H, s, H-6), 5.12 (1H, bs, HO-C4), 3.81 (3H, s, CH_3_O), 2.65–2.45 (2H, m, CH_2_-C2), 1.93–1.91 (1H, m, H-3′), 1.65–1.64 (1H, m, H-4′), 1.65–1.63 (1H, m, H-1′), 1.60–1.56 (1H, m, H-3′), 1.60–1.56 (1H, m, H-4′), 1.49–1.46 (1H, m, H-5′), 1.32–1.30 (1H, m, H-5′), 1.20 (3H, s, CH_3_-C2′), 0.98 (3H, s, CH_3_-C6′), 0.89 (3H, s, CH_3_-C6′); ^13^C NMR (CDCl_3_, 100.6 MHz) δ 146.2 (C-1), 145.3 (C-5), 138.9 (C-4), 114.3 (C-3), 114.2 (C-2), 100.3 (C-6), 76.7 (C-2′), 55.9 (CH_3_O-C-5), 48.3 (C-1′), 41.5 (C-5′), 40.0 (C3′), 33.4 (C-6′), 32.1 (CH_3_-C6′), 22.7 (CH_2_-C-2), 20.7 (CH_3_-C6′), 19.8 (C-4′), 19.6 (CH_3_-C2′); MS *m*/*z* (%) 276 (54.9: M^+^-H_2_O), 191 (18.2), 153 (100), 69 (8.5), 41 (7.3). Fraction IV: Compound **26** (20 mg, 18.9% yield) obtained as a brown viscous oil. IR (cm^−1^) 3396, 2968, 2937, 1646, 1603, 1521, 1446, 1374, 1274, 1196, 1018, 867; ^1^H-NMR (CDCl_3_, 400.1 MHz) δ 6.67 (1H, s, H-3), 6.42 (1H, s, H-6), 5.29 (1H, m, H2′), 3.82 (3H, s, CH_3_O), 3.26 (2H, d, *J* = 7.1 Hz H-1′), 2.07–2.03 (2H, m, H-4′), 1.76 (3H, s, CH_3_-C3′), 1.52–1.48 (2H, m, H-6′), 1.46–1.41 (2H, m, H-5′), 1.22 (6H, s, CH_3_-C7′ and H-8′); ^12^C NMR (CDCl_3_, 100.6 MHz) δ 147.4 (C-1), 145.4 (C-5), 139.3 (C-4), 138.3 (C-3′), 122.0 (C-2′), 118.5 (C-2), 115.3 (C-3), 100.4 (C-6), 70.9 (C-7′), 56.1 (CH_3_O-C5), 43.3 (C-6′), 39.9 (C-4′), 29.2 (CH_3_-C7′ and C8′), 28.9 (C-1′), 22.5 (C-5′), 16.1 (CH_3_-C3′); MS *m*/*z* (%) 292 (8.9: M^+^-H_2_), 277 (3.6), 219 (1.8), 203 (8.9), 191 (100: M^+^-H_2_-101 (C_6_H_13_O)), 176 (8.9), 148 (3.6), 91 (1.8), 69 (1.8), 43 (1.8). Fraction V: Compound **21** (12 mg, 10.7% yield) obtained as brown viscous oil. The spectroscopic data (IR, MS and NMR) were consistent with those previously reported [27].

### 3.3. In Vitro Effect of the Compounds on the Mycelial Growth of B. cinerea

The antifungal activities of all tested compounds were evaluated using the radial growth test at final concentrations of 50, 150 and 250 mg/L in PDA medium [37]. Captan was used as the positive control, whereas PDA medium containing 1% ethanol was considered as the negative control. The percentages of inhibitions were determined following a standard method [42]. Experimental conditions have been detailed elsewhere [30].

## 4. Conclusions

Hydrated geranylphenols were synthesized by following two different synthetic pathways: direct coupling of geraniol with *o*-cresol in dioxane with added water and using BF_3_·Et_2_O as the catalyst; or by the reaction of a geranylated phenol with BF_3_·Et_2_O in dioxane and added water. Interestingly, the coupling of geraniol to hydroquinones gives completely different products.

On the other hand, the mycelial growth inhibition of hydrated geranylphenols is in the range of 30%–95% at 250 ppm. The percentages of inhibition induced by hydrated compounds (**23** and **26**) are higher than those produced by the respective geranylated phenol (**14** and **17**). The enhancement of the antifungal activity is larger for hydrated compounds carrying only one hydroxyl group in the side chain (**25** and **26**) than for completely hydrated compounds (**22**–**24**). It is worth stressing that the new Compound **26** exhibits antifungal activity similar to Captan, a common fungicide used to control *B. cinerea*. Finally, as far as we know, this is the first study relating the structure of the geranyl chain with the antifungal activity of geranylated phenols.

## Figures and Tables

**Table 1:** Percentage of inhibition of geranylated phenols (**14**–**18**), geranylated quinones (**19**–**21**) and hydrated geranylphenols (**22**–**26**) on the mycelial growth of *B. cinerea* strain GM7 at 72 h *in vitro*.

Compounds	Percentage of Inhibition on Mycelial Growth of *B. cinerea in Vitro* (%)		
	50 mg/L	150 mg/L	250 mg/L
**14**	0 ± 0	0 ± 0	0 ± 0
**15**	9 ± 4	6 ± 3	8 ± 5
**16**	0 ± 0	0 ± 0	9 ± 0
**17**	49 ± 2	56 ± 2	56 ± 0
**18**	36 ± 3	48 ± 3	52 ± 2
**19**	30 ± 2	51 ± 1	69 ± 1
**20**	43 ± 8	58 ± 8	73 ± 8
**21**	36 ± 0	64 ± 0	75 ± 0
**22**	0 ± 0	30 ± 2	53 ± 3
**23**	0 ± 0	0 ± 0	28 ± 7
**24**	36 ± 0	66 ± 4	67 ± 5
**25**	50 ± 6	81 ± 5	90 ± 1
**26**	81 ± 0	91 ± 0	94 ± 0
**C− ^1^**	0 ± 0	0 ± 0	0 ± 0
**C+ ^2^**	94 ± 5	94 ± 0	99 ± 0

The percentage of inhibition of mycelial growth is based on colony diameter measurements after 72 h of incubation. Each point represents the mean of at least three independent experiments ± the standard deviation. ^1^ C− refers to the negative control; and ^2^ C+ refers to the positive control (Captan).

## Supplementary Materials

Supplementary materials can be found at http://www.mdpi.com/1422-0067/17/6/840/s1.
