# Supplementary material for: Synthesis of New Hydrated Geranylphenols and in Vitro Antifungal Activity against Botrytis cinerea
Source: Int J Mol Sci. 2016 Jun 3;17(6):840. doi: 10.3390/ijms17060840 (PMC4926374; doi:10.3390/ijms17060840)
Supplement: Supplementary file 1 [file ijms-17-00840-s001.pdf]

Supplementary Materials: Synthesis of New Hydrated Geranylphenols and *in Vitro* Antifungal Activity against *Botrytis cinerea*

Mauricio Soto, Luis Espinoza, María I. Chávez, Katy Díaz, Andrés F. Olea and Lautaro Taborga

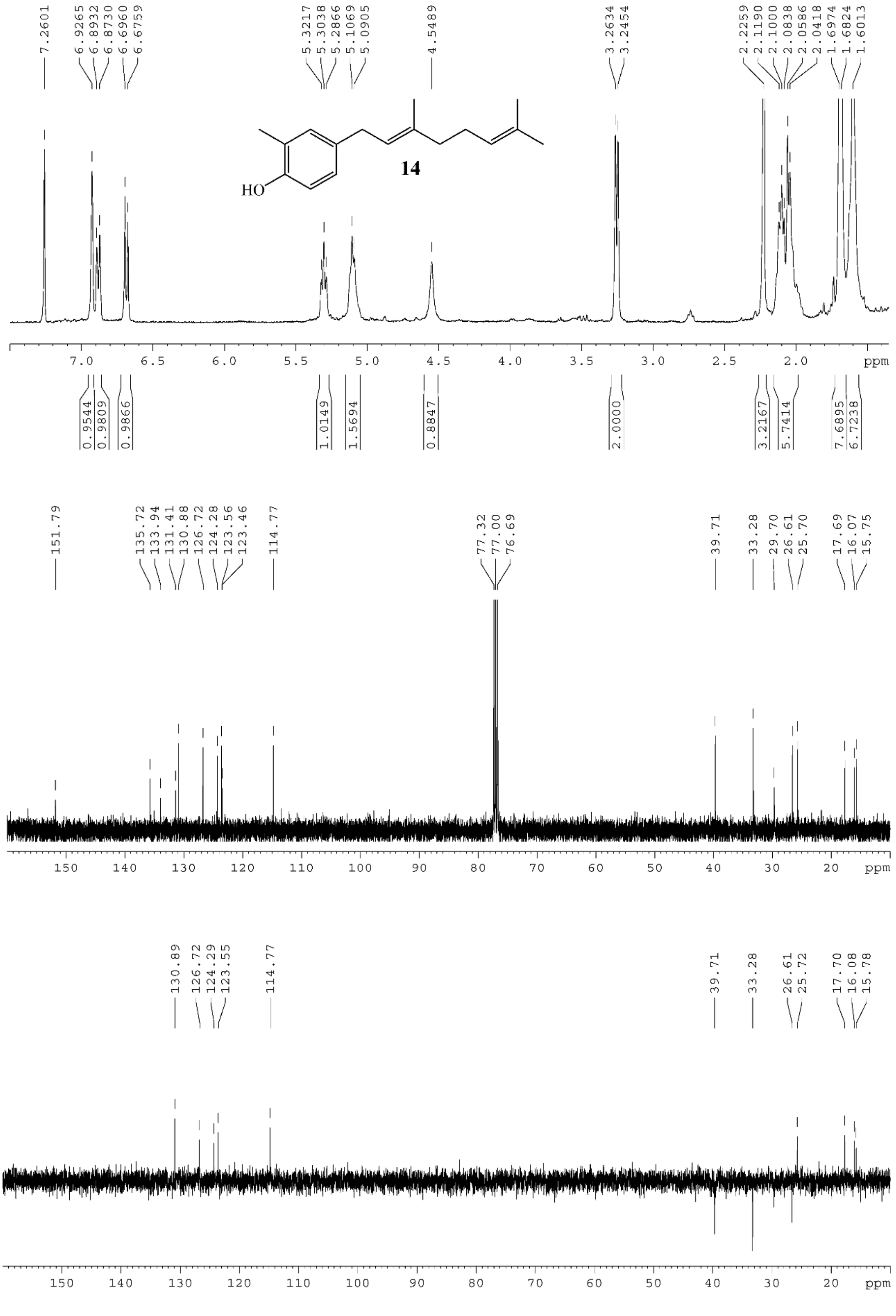

Figure S1. *Cont.*

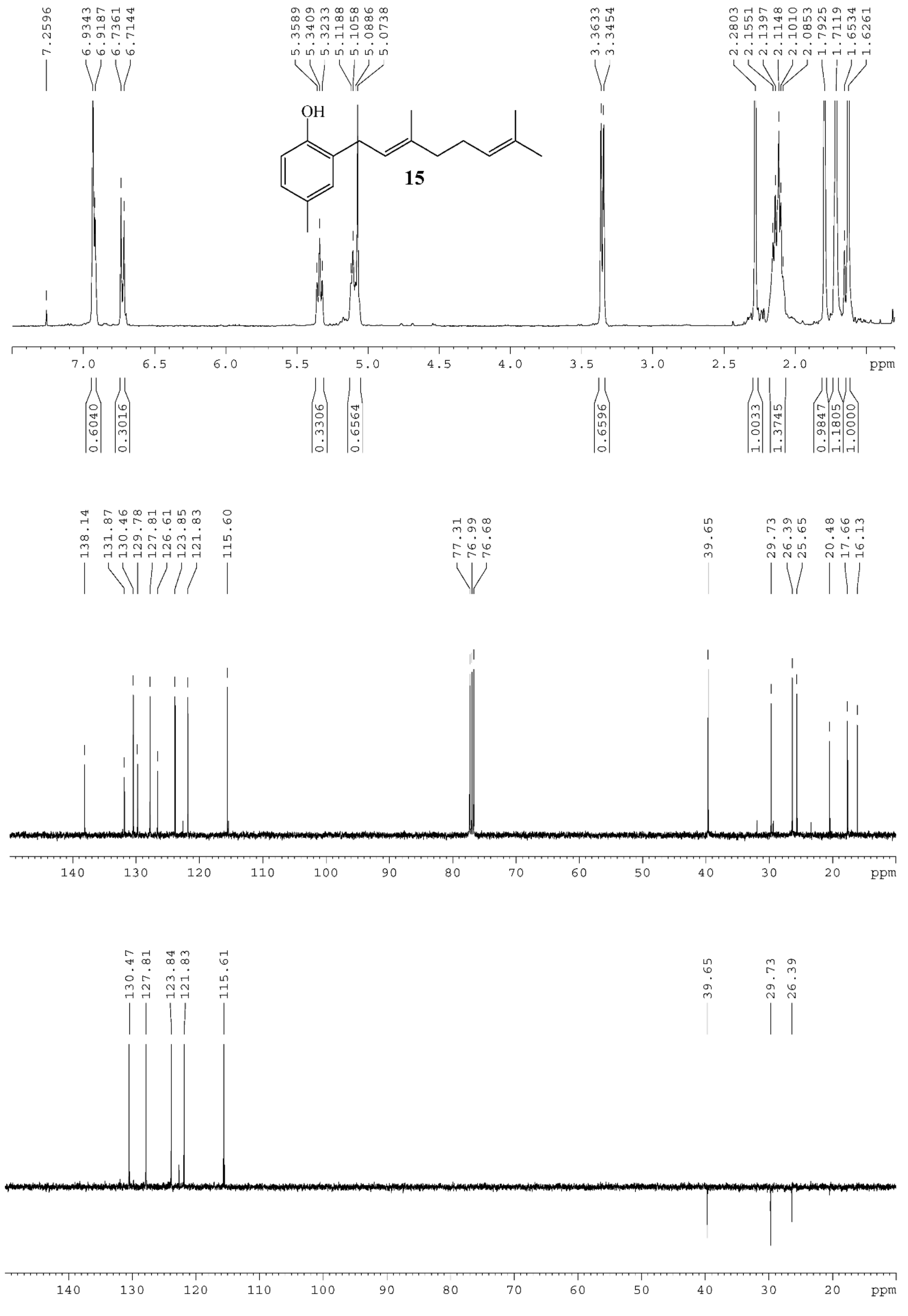

Figure S1. *Cont.*

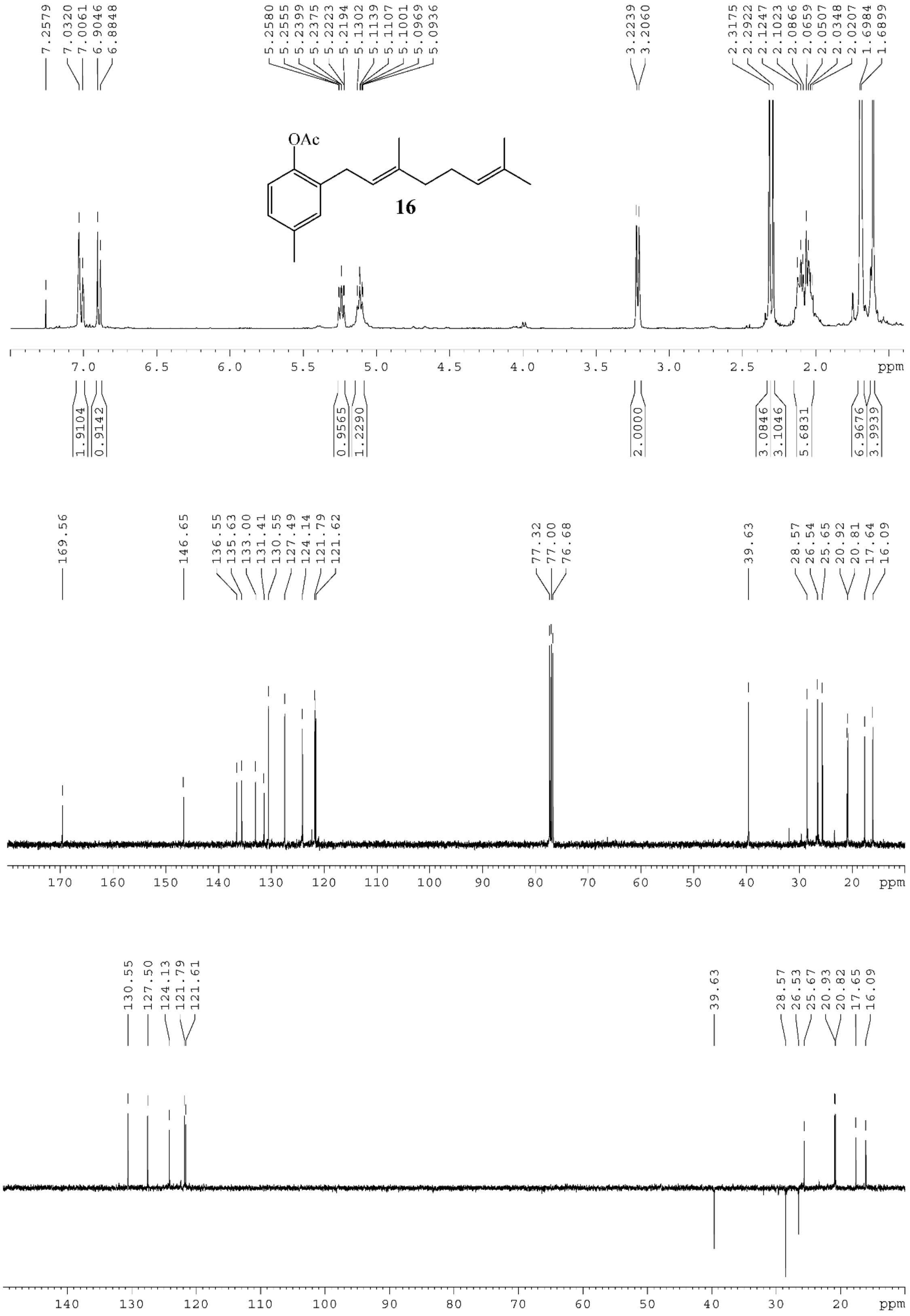

Figure S1. *Cont.*

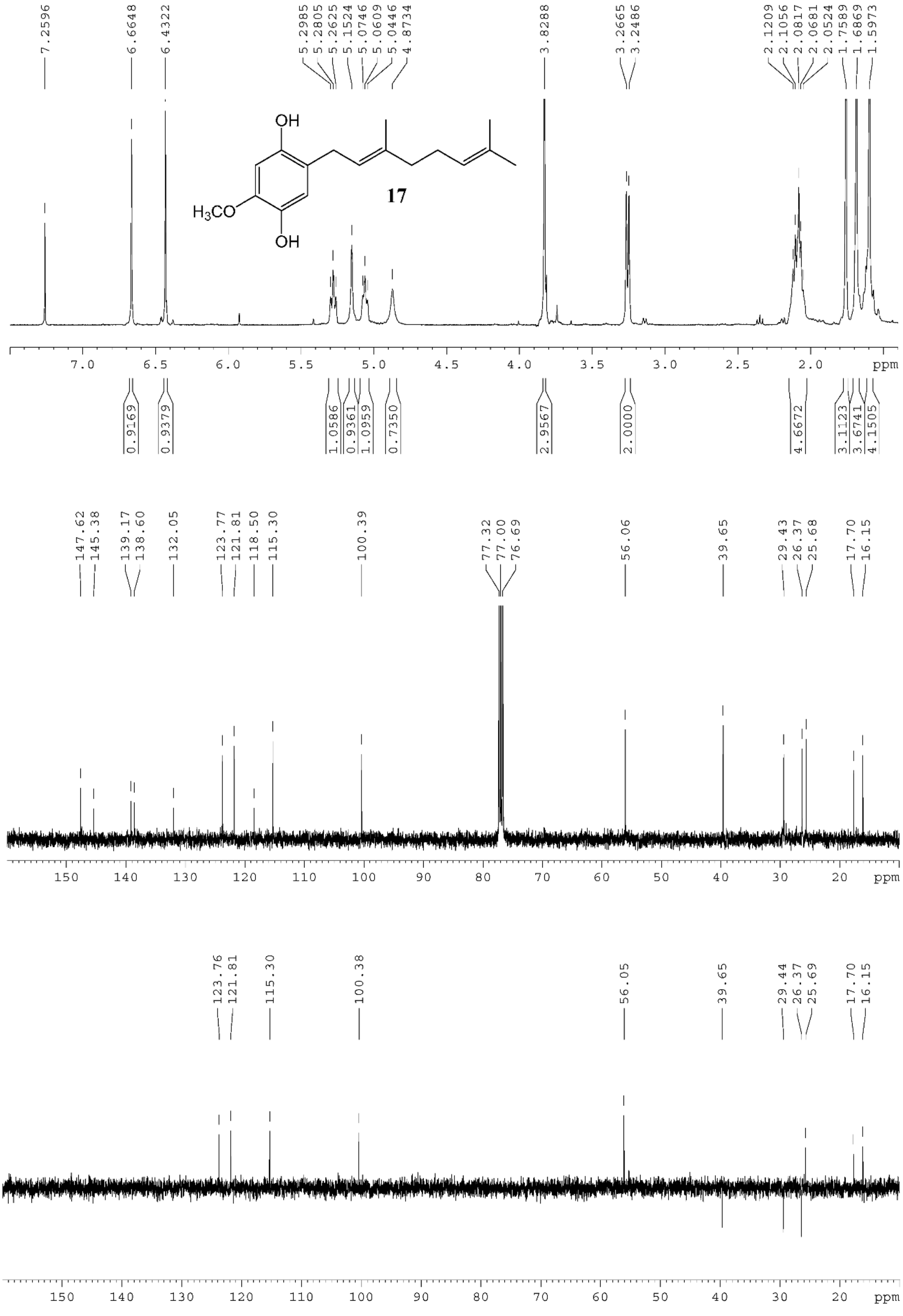

Figure S1. *Cont.*

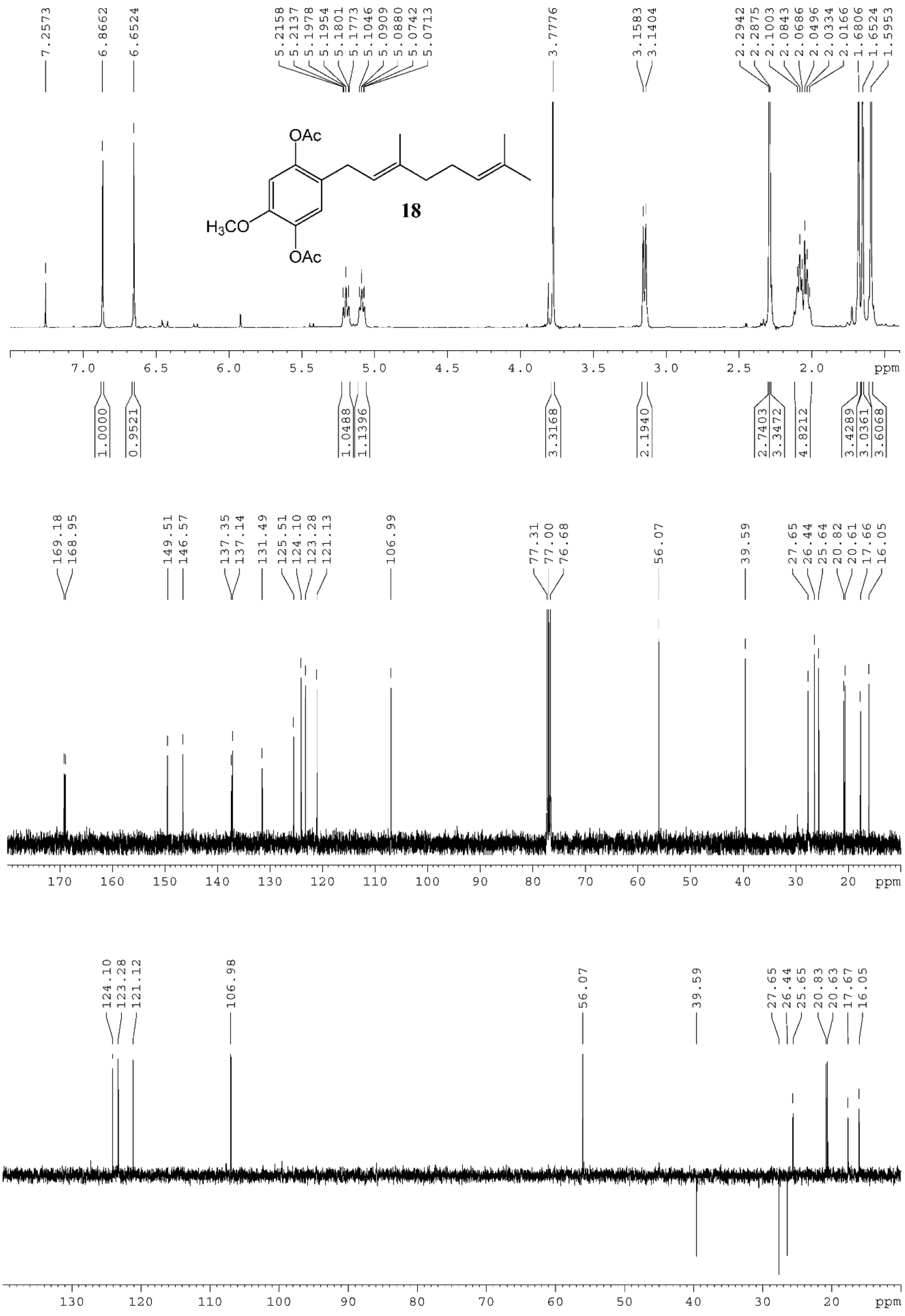

Figure S1. *Cont.*

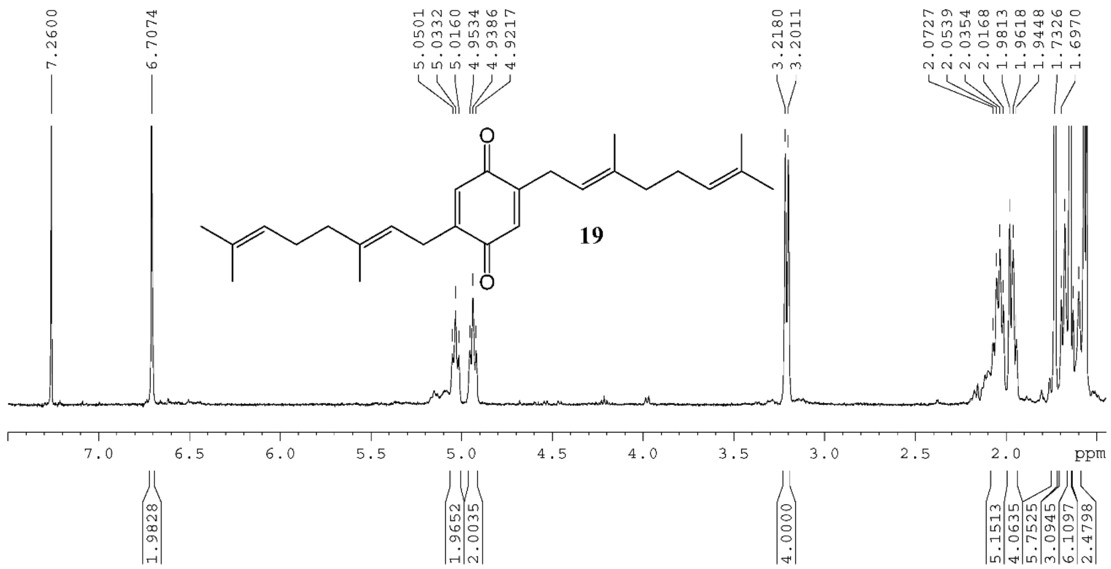

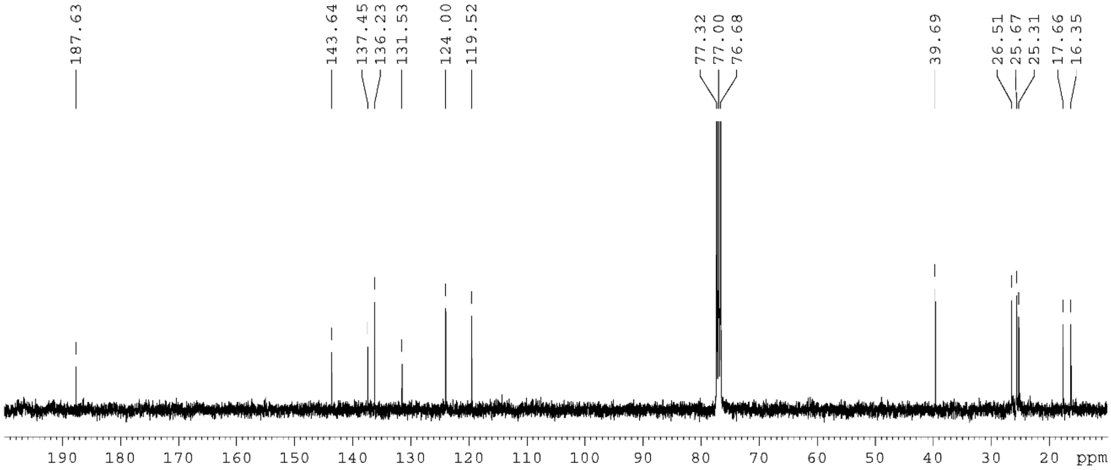

Figure S1. *Cont.*

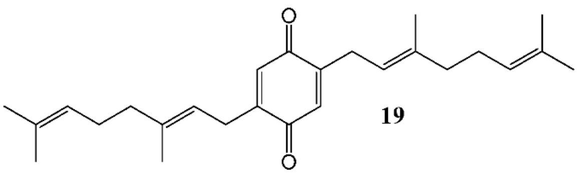

19

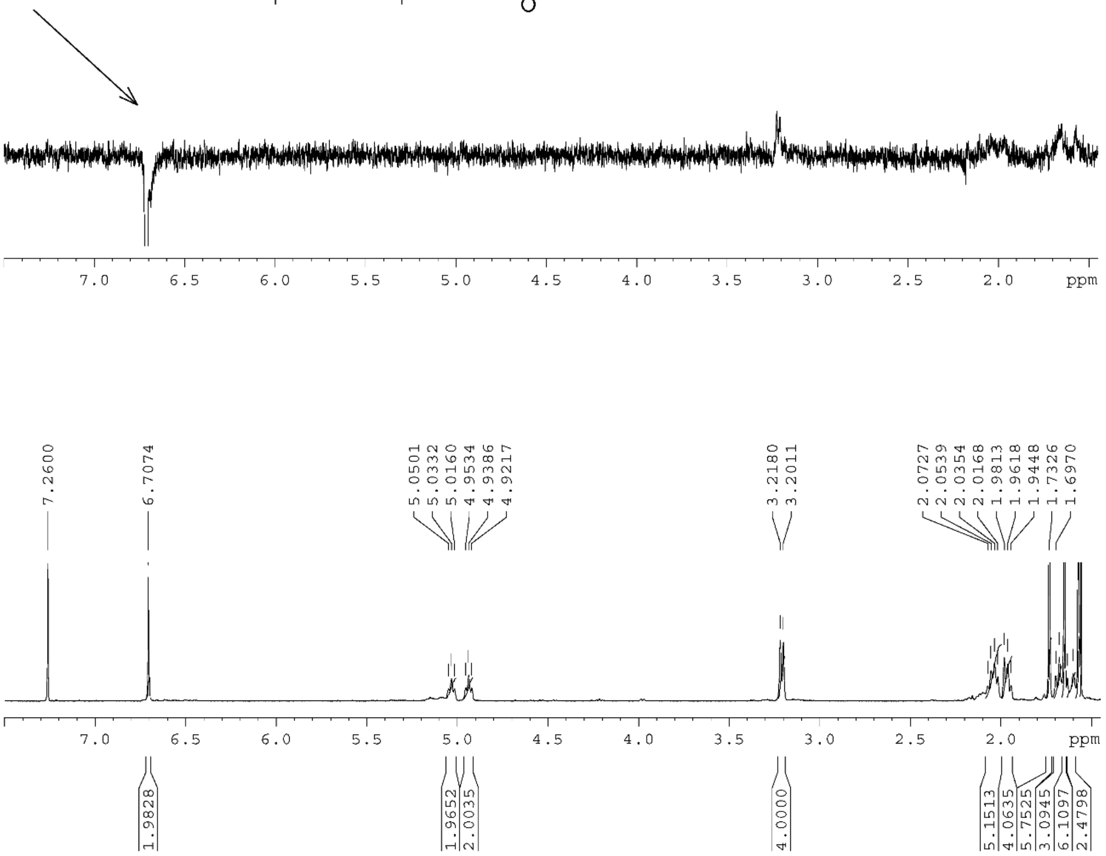

Figure S1. *Cont.*

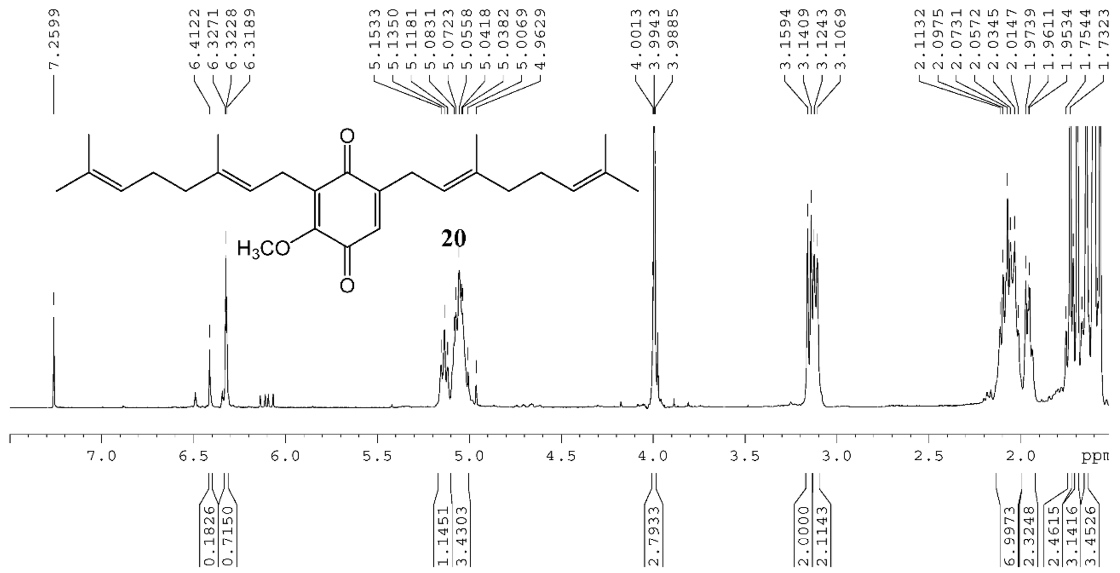

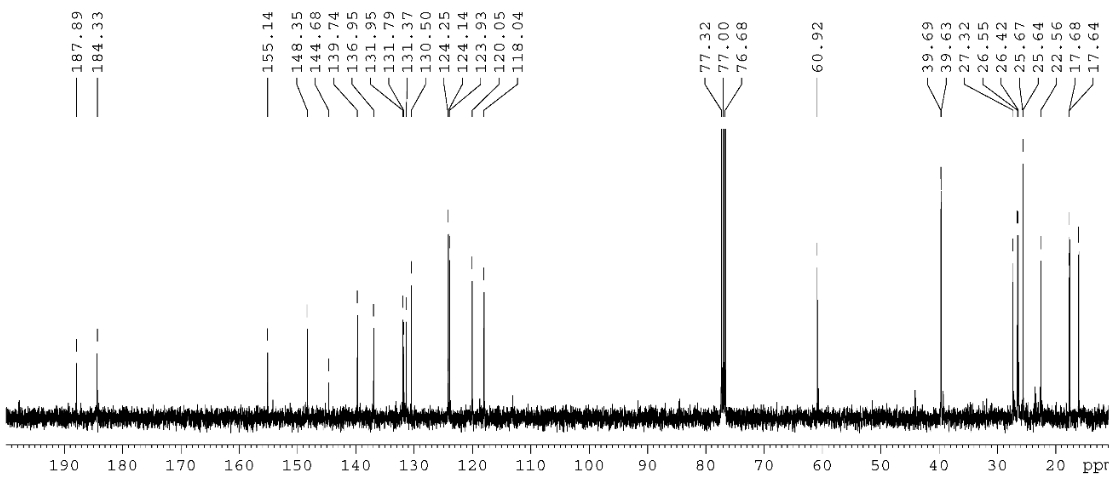

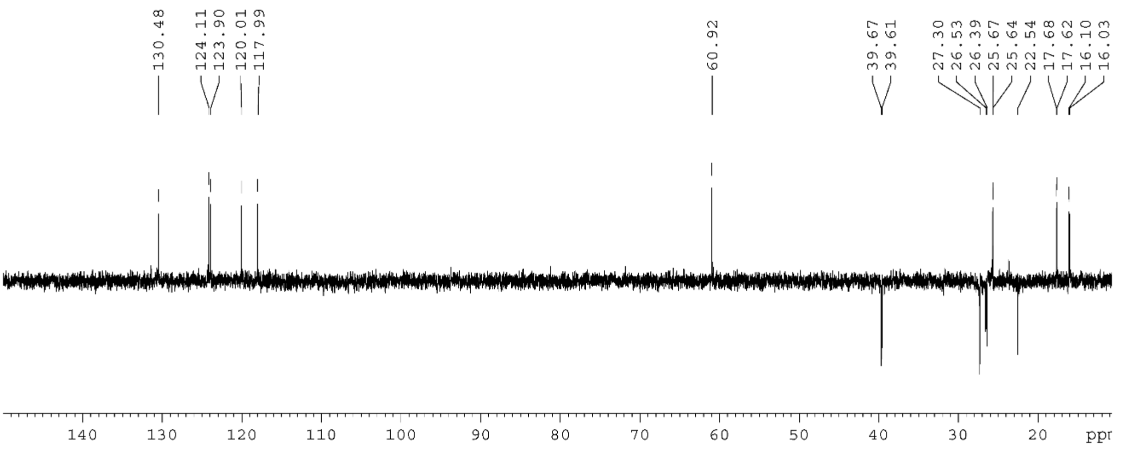

Figure S1. *Cont.*

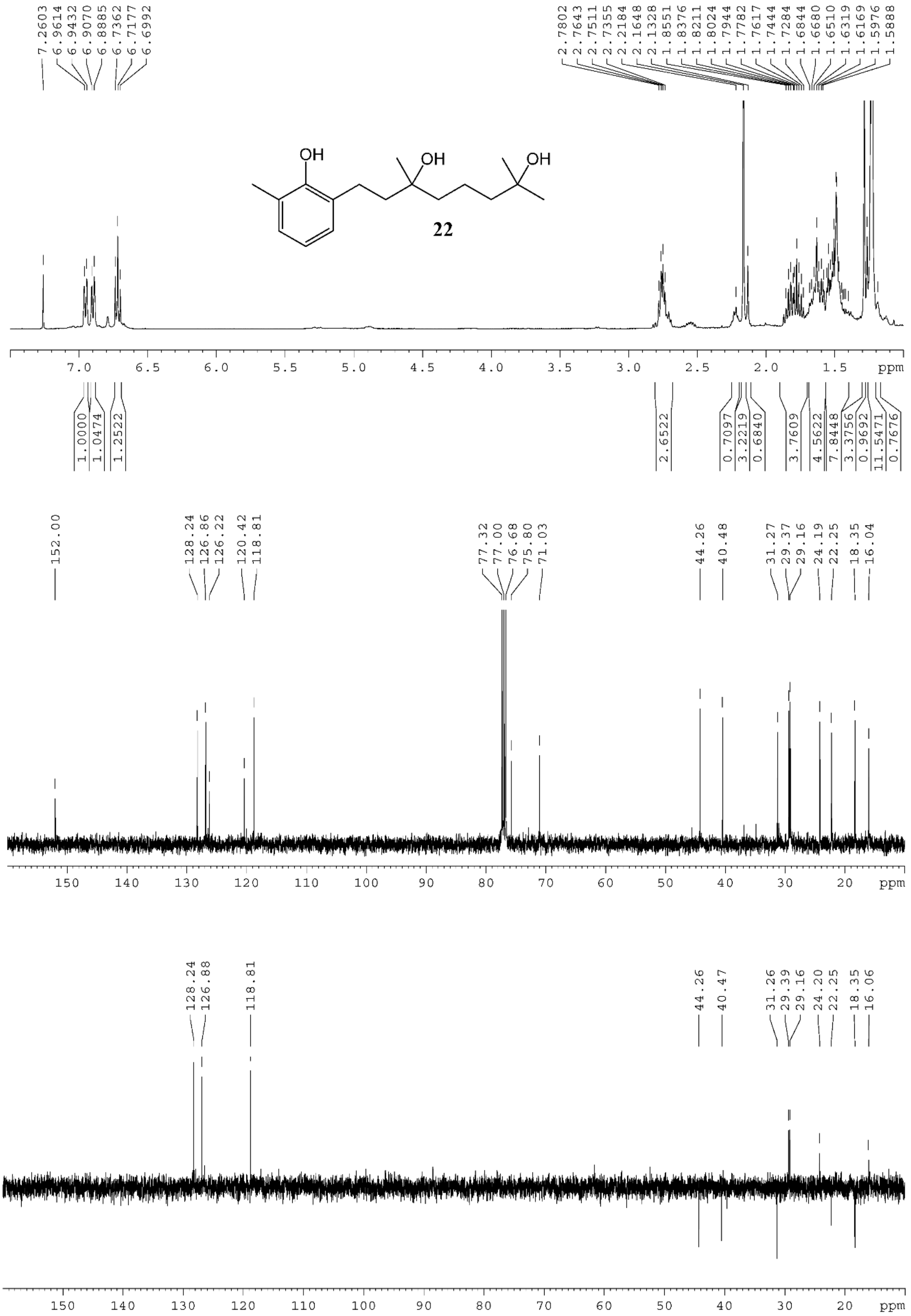

Figure S1. *Cont.*

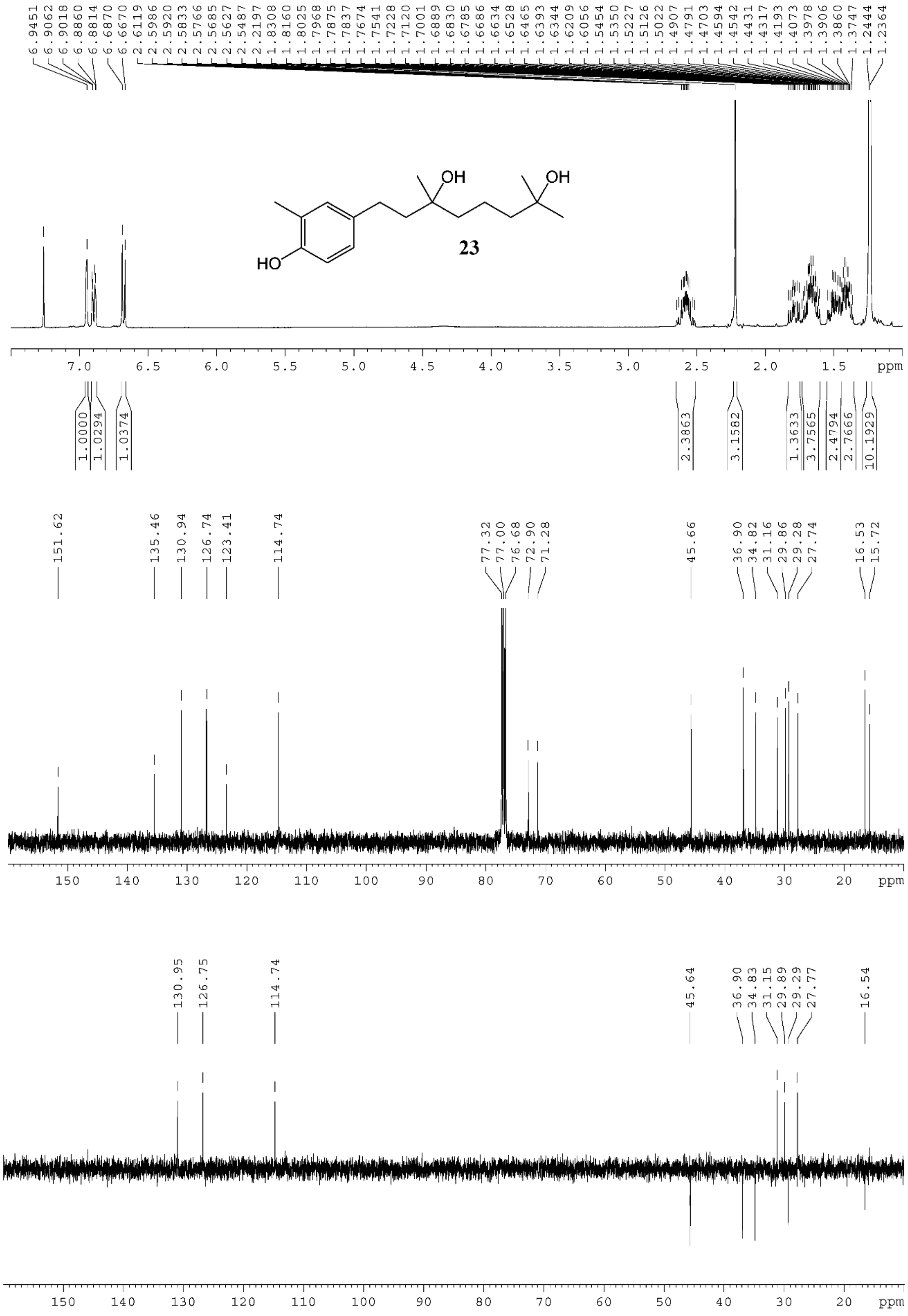

Figure S1. *Cont.*

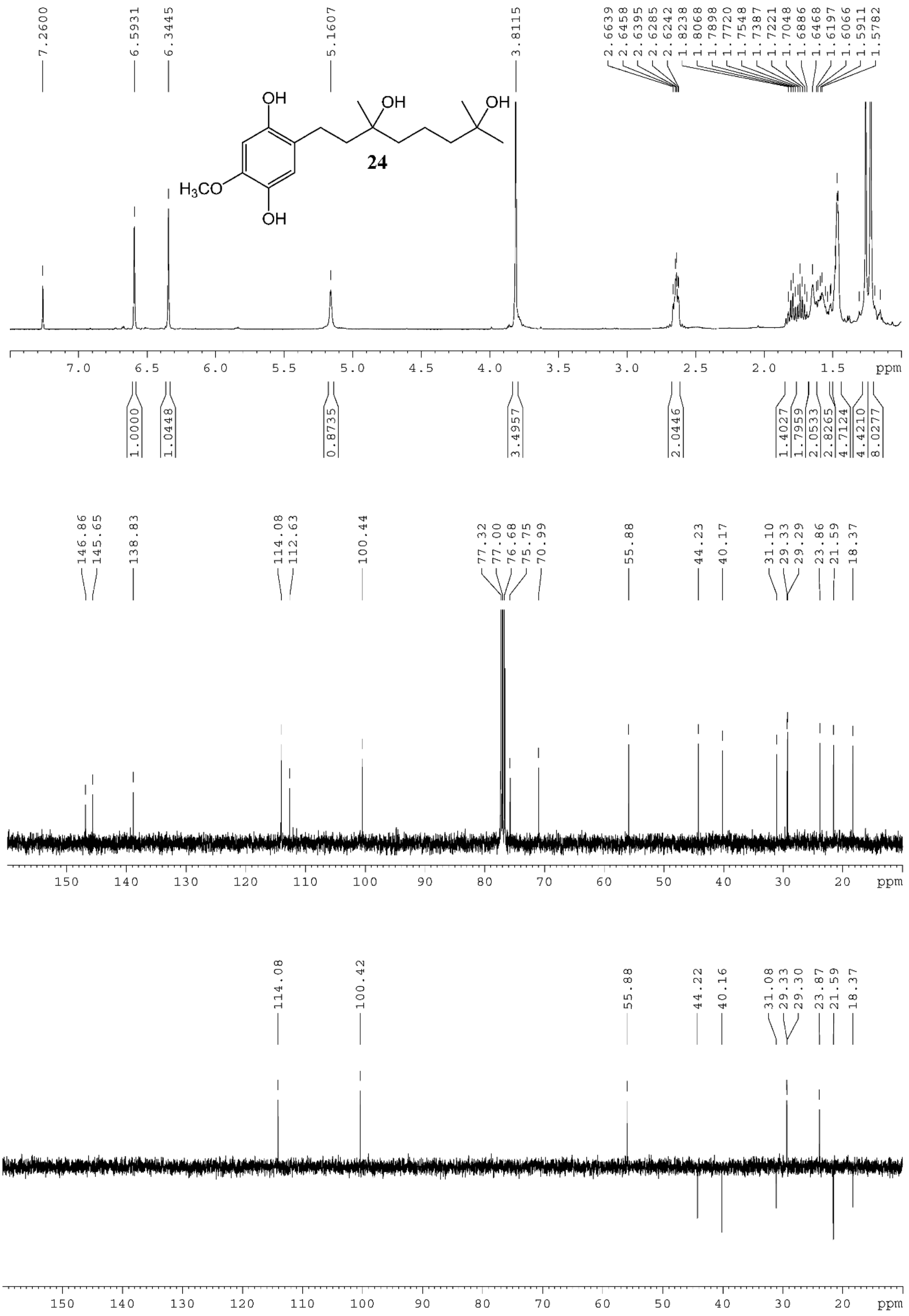

Figure S1. *Cont.*

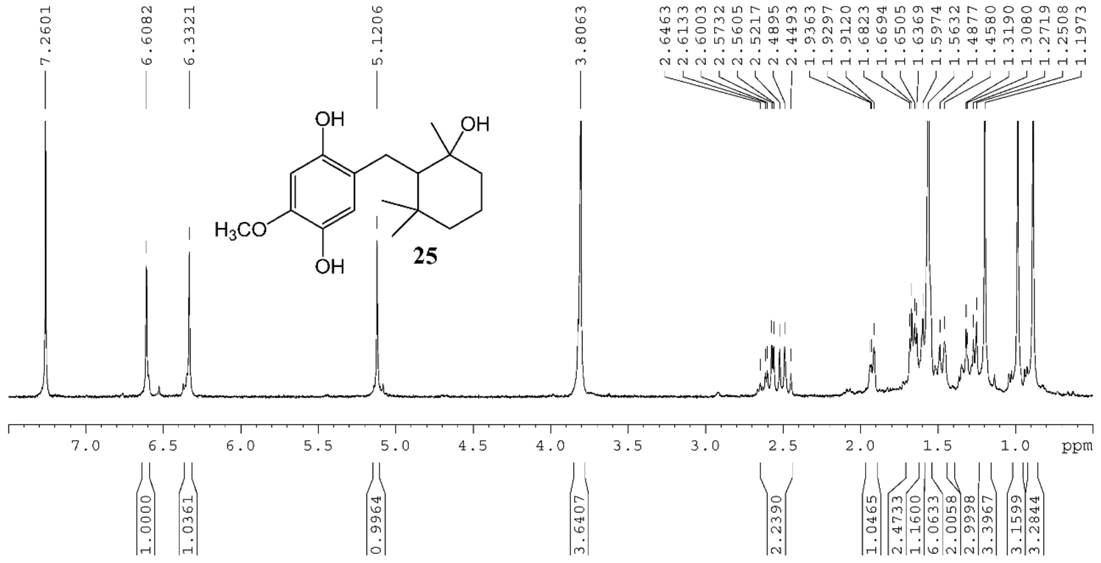

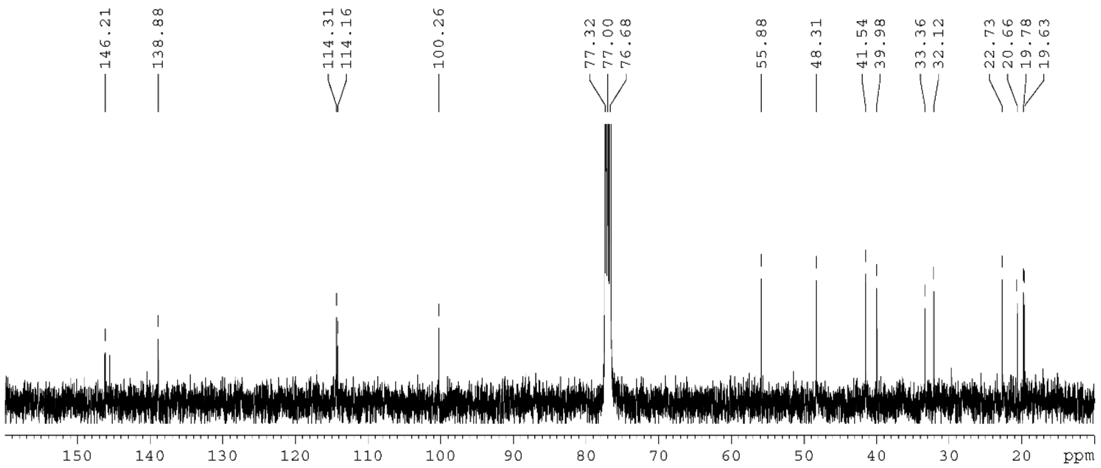

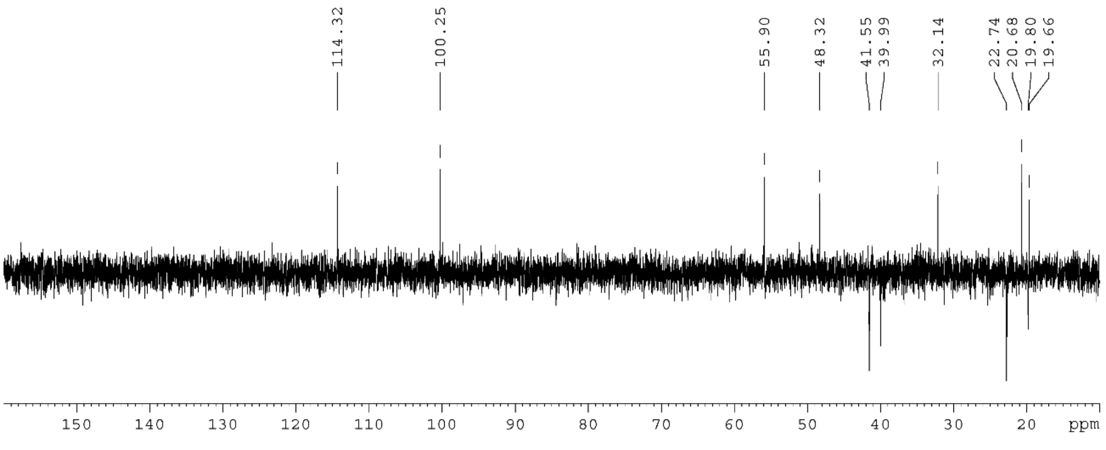

Figure S1. *Cont.*

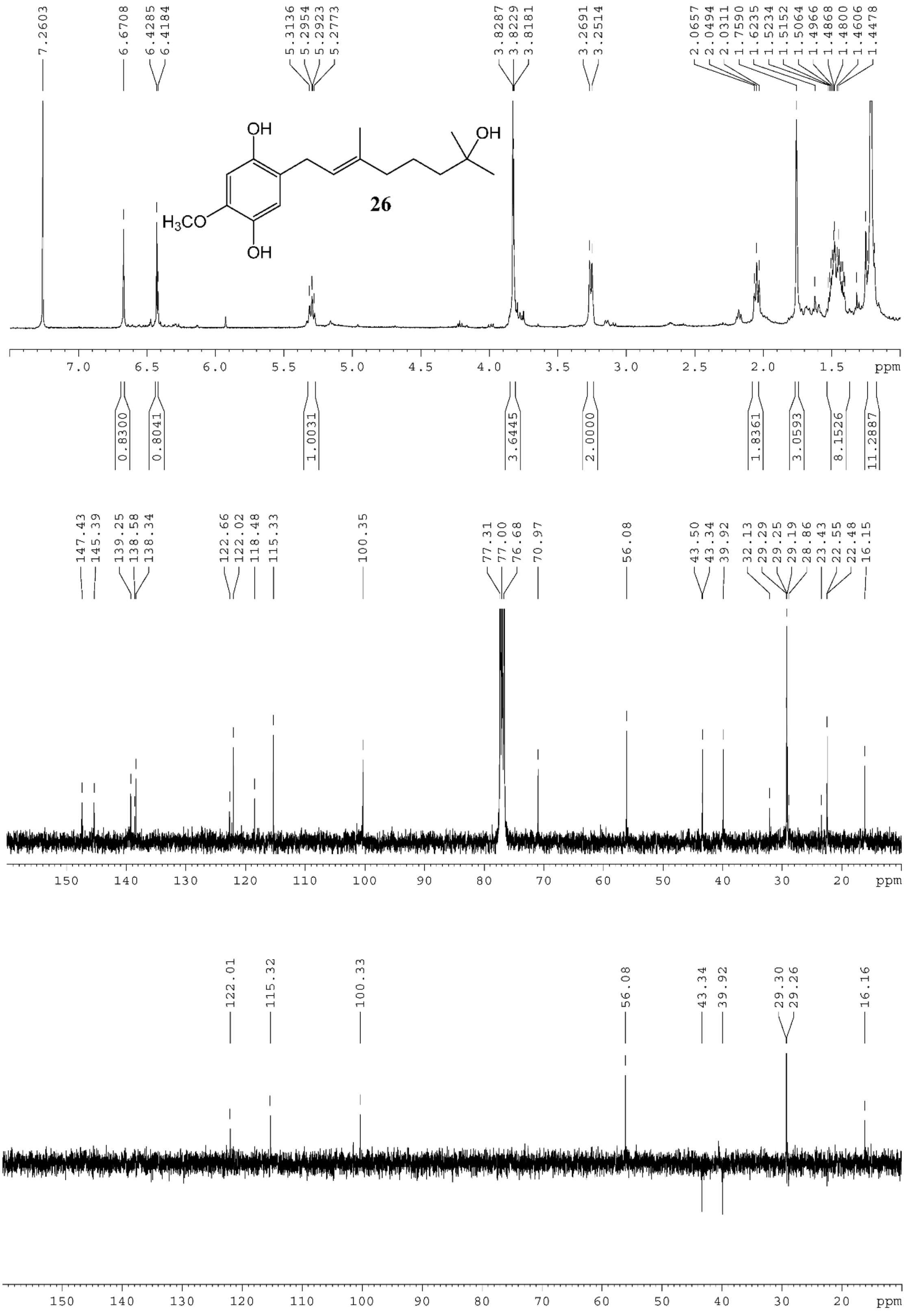

Figure S1. NMR spectra of compounds **14–20**, **22–26**.
